# Supplementary material for: Prioritizing long COVID related single nucleotide polymorphisms by mining genome-wide association studies of COVID-19 susceptibility and hospitalization
Source: Front Syst Biol. 2026 Jun 5;6:1797543. doi: 10.3389/fsysb.2026.1797543 (PMC13279042; doi:10.3389/fsysb.2026.1797543)
Supplement: Supplementary file 1 [file DataSheet1.zip › SuppFiguresTables4Frontiers/SupplementaryFigures1-24.pdf]

Figure S1

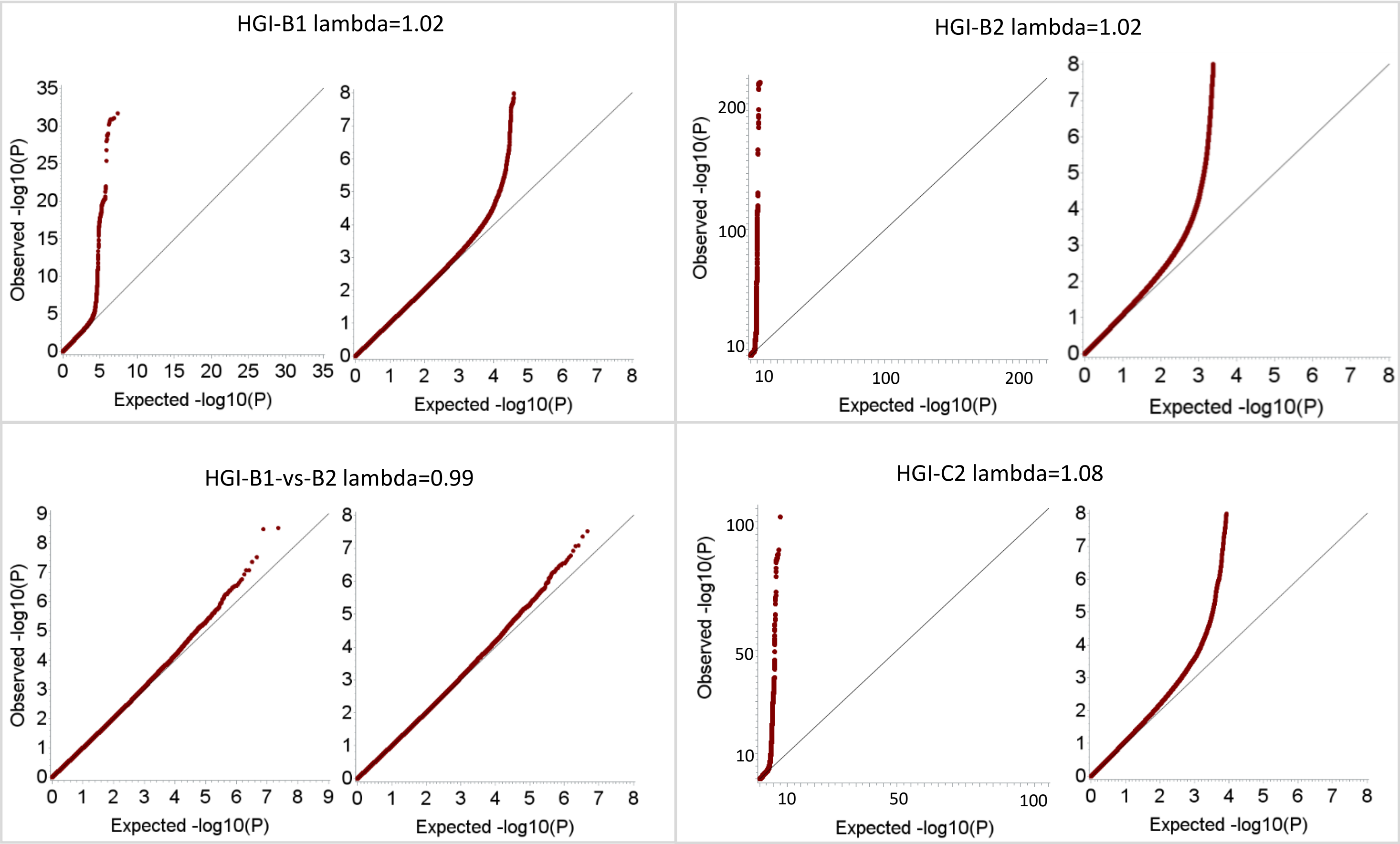
HGI-B1 lambda=1.02
HGI-B2 lambda=1.02
HGI-B1-vs-B2 lambda=0.99
HGI-C2 lambda=1.08
Observed -log10(P)
Expected -log10(P)

Figure S2

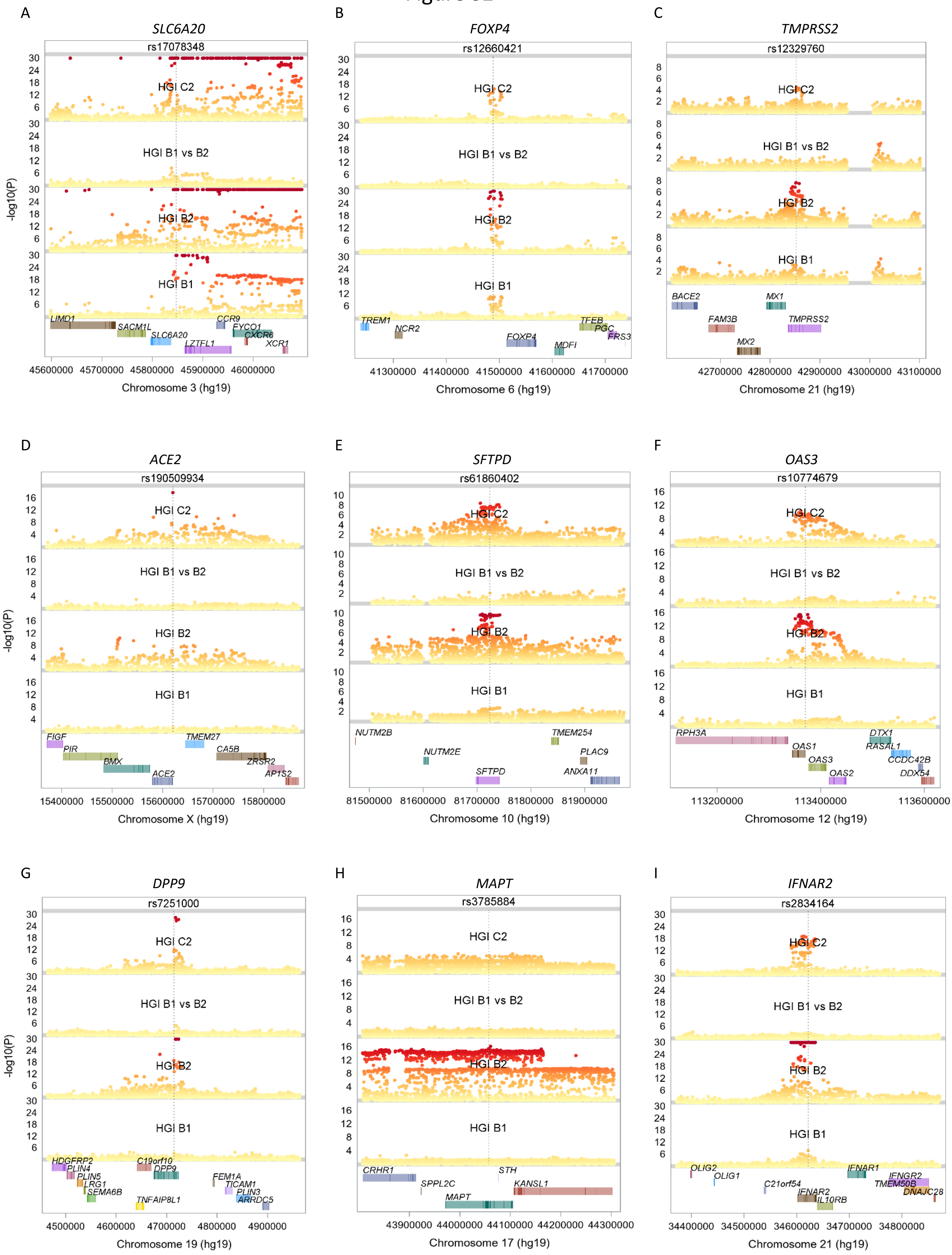
A
SLC6A20
rs17078348
HGI C2
HGI B1 vs B2
HGI B2
HGI B1
-log10(P)
LIMD1
SACM1L
SLC6A20
CCR9
FYCO1
CXCR6
LZTFL1
XCR1
Chromosome 3 (hg19)
B
FOXP4
rs12660421
HGI C2
HGI B1 vs B2
HGI B2
HGI B1
TREM1
NCR2
FOXP4
MDFI
TFEB
PGC
FRS3
Chromosome 6 (hg19)
C
TMPRSS2
rs12329760
HGI C2
HGI B1 vs B2
HGI B2
HGI B1
BACE2
MX1
FAM3B
TMPRSS2
MX2
Chromosome 21 (hg19)
D
ACE2
rs190509934
HGI C2
HGI B1 vs B2
HGI B2
HGI B1
-log10(P)
FIGF
PIR
BMX
ACE2
TMEM27
CA5B
ZRSR2
AP1S2
Chromosome X (hg19)
E
SFTPD
rs61860402
HGI C2
HGI B1 vs B2
HGI B2
HGI B1
NUTM2B
NUTM2E
SFTPD
TMEM254
PLAC9
ANXA11
Chromosome 10 (hg19)
F
OAS3
rs10774679
HGI C2
HGI B1 vs B2
HGI B2
HGI B1
RPH3A
OAS1
OAS3
OAS2
DTX1
RASAL1
CCDC42B
DDX54
Chromosome 12 (hg19)
G
DPP9
rs7251000
HGI C2
HGI B1 vs B2
HGI B2
HGI B1
-log10(P)
HDGFRP2
PLIN4
PLIN5
LRG1
SEMA6B
C19orf10
DPP9
TNFAIP8L1
FEM1A
TICAM1
PLIN3
ARRDC5
Chromosome 19 (hg19)
H
MAPT
rs3785884
HGI C2
HGI B1 vs B2
HGI B2
HGI B1
CRHR1
SPPL2C
MAPT
STH
KANSL1
Chromosome 17 (hg19)
I
IFNAR2
rs2834164
HGI C2
HGI B1 vs B2
HGI B2
HGI B1
OLIG2
OLIG1
C21orf54
IFNAR2
IL10RB
IFNAR1
IFNGR2
TMEM50B
DNAJC28
Chromosome 21 (hg19)

Figure S3

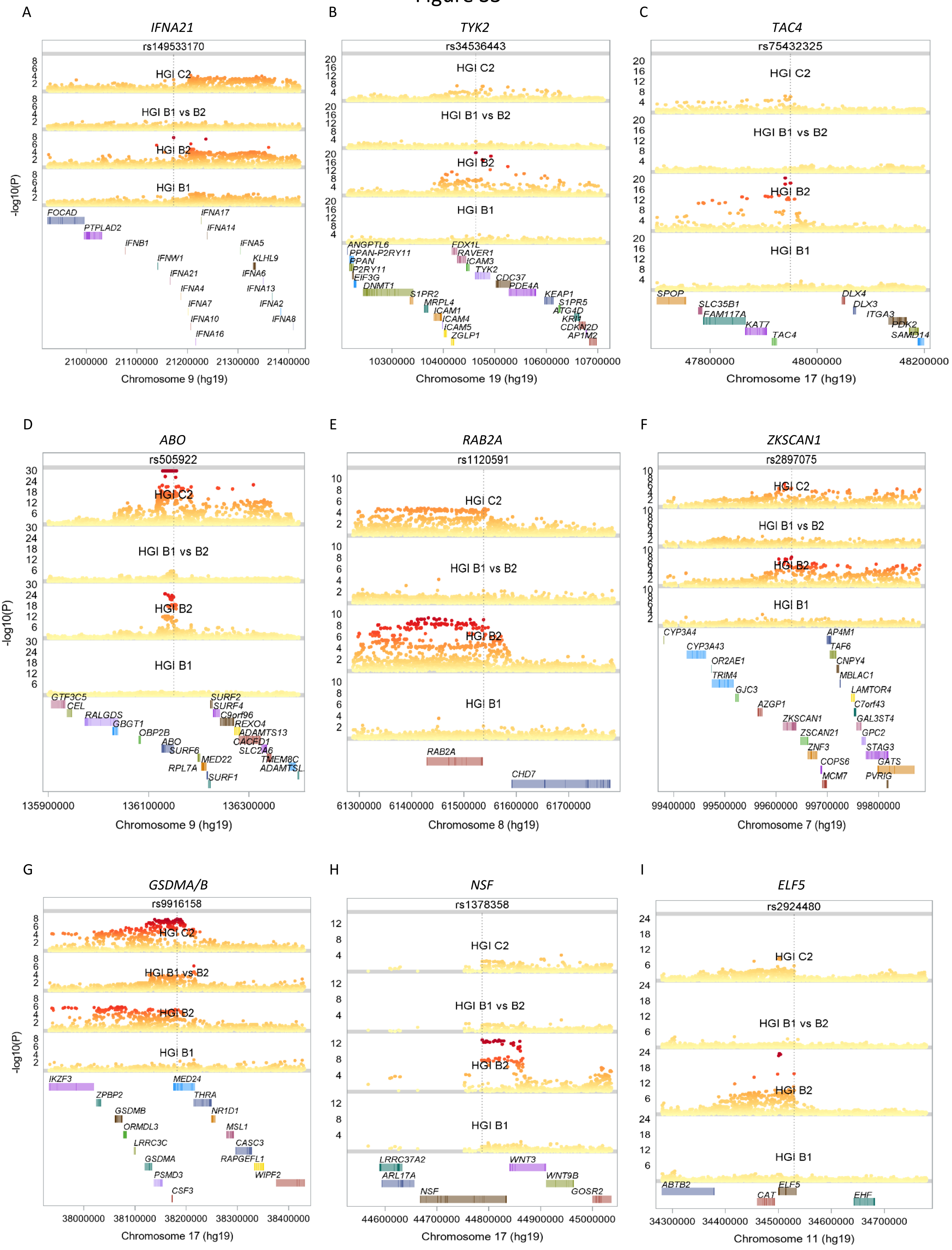

A
IFNA21
rs149533170
HGI C2
HGI B1 vs B2
HGI B2
HGI B1
-log10(P)
Chromosome 9 (hg19)
B
TYK2
rs34536443
Chromosome 19 (hg19)
C
TAC4
rs75432325
Chromosome 17 (hg19)
D
ABO
rs505922
Chromosome 9 (hg19)
E
RAB2A
rs1120591
Chromosome 8 (hg19)
F
ZKSCAN1
rs2897075
Chromosome 7 (hg19)
G
GSDMA/B
rs9916158
Chromosome 17 (hg19)
H
NSF
rs1378358
Chromosome 17 (hg19)
I
ELF5
rs2924480
Chromosome 11 (hg19)

Figure S4

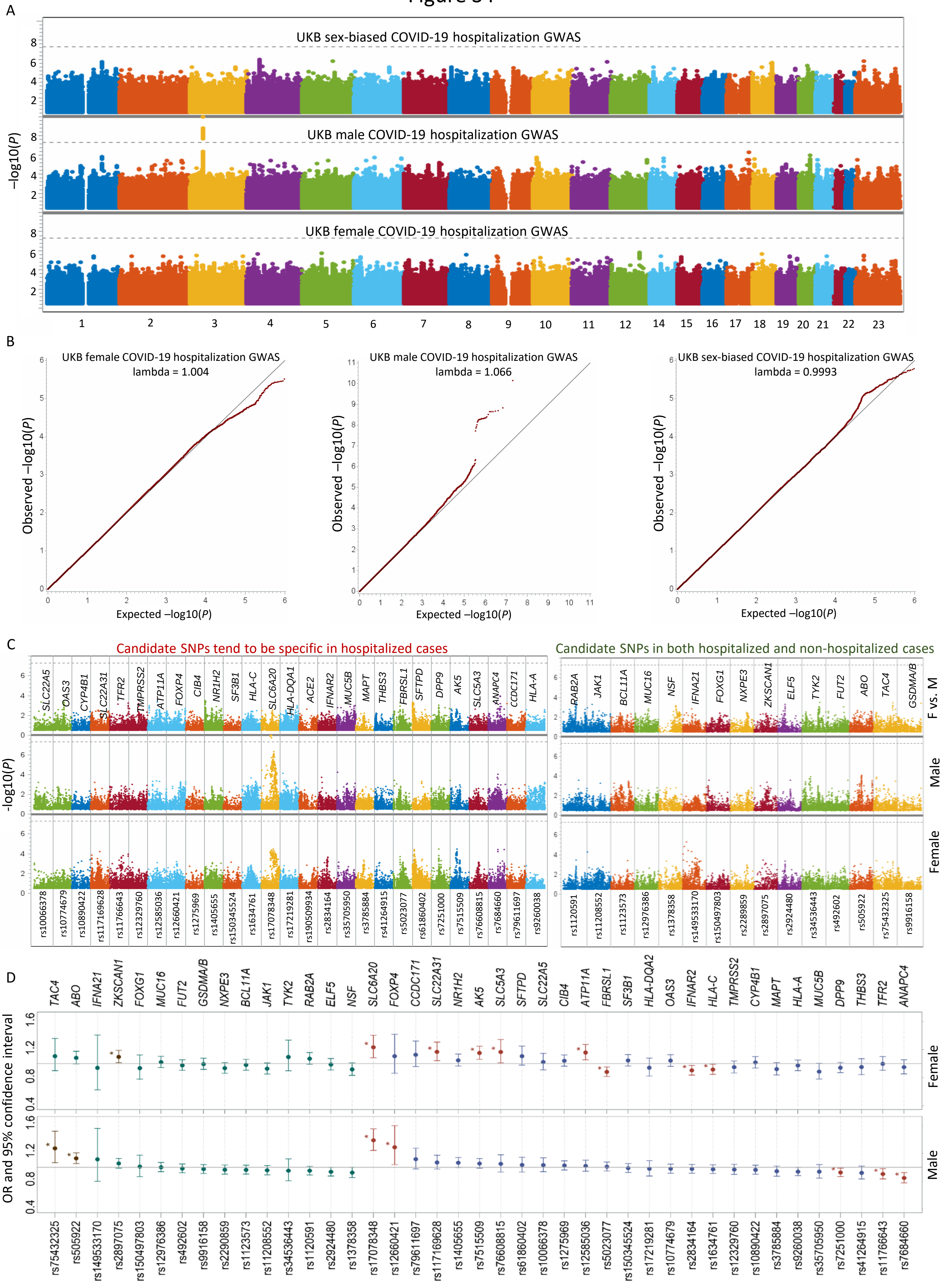

A
UKB sex-biased COVID-19 hospitalization GWAS
UKB male COVID-19 hospitalization GWAS
UKB female COVID-19 hospitalization GWAS
−log10(P)
1 2 3 4 5 6 7 8 9 10 11 12 14 15 16 17 18 19 20 21 22 23
B
UKB female COVID-19 hospitalization GWAS
lambda = 1.004
UKB male COVID-19 hospitalization GWAS
lambda = 1.066
UKB sex-biased COVID-19 hospitalization GWAS
lambda = 0.9993
Observed −log10(P)
Expected −log10(P)
C
Candidate SNPs tend to be specific in hospitalized cases
Candidate SNPs in both hospitalized and non-hospitalized cases
SLC22A5 OAS3 CYP4B1 SLC22A31 TFR2 TMPRSS2 ATP11A FOXP4 CIB4 NR1H2 SF3B1 HLA-C SLC6A20 HLA-DQA1 ACE2 IFNAR2 MUC5B MAPT THBS3 FBRSL1 SFTPD DPP9 AK5 SLC5A3 ANAPC4 CCDC171 HLA-A
RAB2A JAK1 BCL11A MUC16 NSF IFNA21 FOXG1 NXPE3 ZKSCAN1 ELF5 TYK2 FUT2 ABO TAC4 GSDMA/B
F vs. M
Male
Female
-log10(P)
rs10066378 rs10774679 rs10890422 rs117169628 rs11766643 rs12329760 rs12585036 rs12660421 rs1275969 rs1405655 rs150345524 rs1634761 rs17078348 rs17219281 rs190509934 rs2834164 rs35705950 rs3785884 rs41264915 rs5023077 rs61860402 rs7251000 rs7515509 rs76608815 rs7684660 rs79611697 rs9260038
rs1120591 rs11208552 rs1123573 rs12976386 rs1378358 rs149533170 rs150497803 rs2289859 rs2897075 rs2924480 rs34536443 rs492602 rs505922 rs75432325 rs9916158
D
TAC4 ABO IFNA21 ZKSCAN1 FOXG1 MUC16 FUT2 GSDMA/B NXPE3 BCL11A JAK1 TYK2 RAB2A ELF5 NSF SLC6A20 FOXP4 CCDC171 SLC22A31 NR1H2 AK5 SLC5A3 SFTPD SLC22A5 CIB4 ATP11A FBRSL1 SF3B1 HLA-DQA2 OAS3 IFNAR2 HLA-C TMPRSS2 CYP4B1 MAPT HLA-A MUC5B DPP9 THBS3 TFR2 ANAPC4
OR and 95% confidence interval
Female
Male
rs75432325 rs505922 rs149533170 rs2897075 rs150497803 rs12976386 rs492602 rs9916158 rs2290859 rs1123573 rs11208552 rs34536443 rs1120591 rs2924480 rs1378358 rs17078348 rs12660421 rs79611697 rs117169628 rs1405655 rs7515509 rs76608815 rs61860402 rs10066378 rs1275969 rs12585036 rs5023077 rs150345524 rs17219281 rs10774679 rs2834164 rs1634761 rs12329760 rs10890422 rs3785884 rs9260038 rs35705950 rs7251000 rs41264915 rs11766643 rs7684660

Figure S5

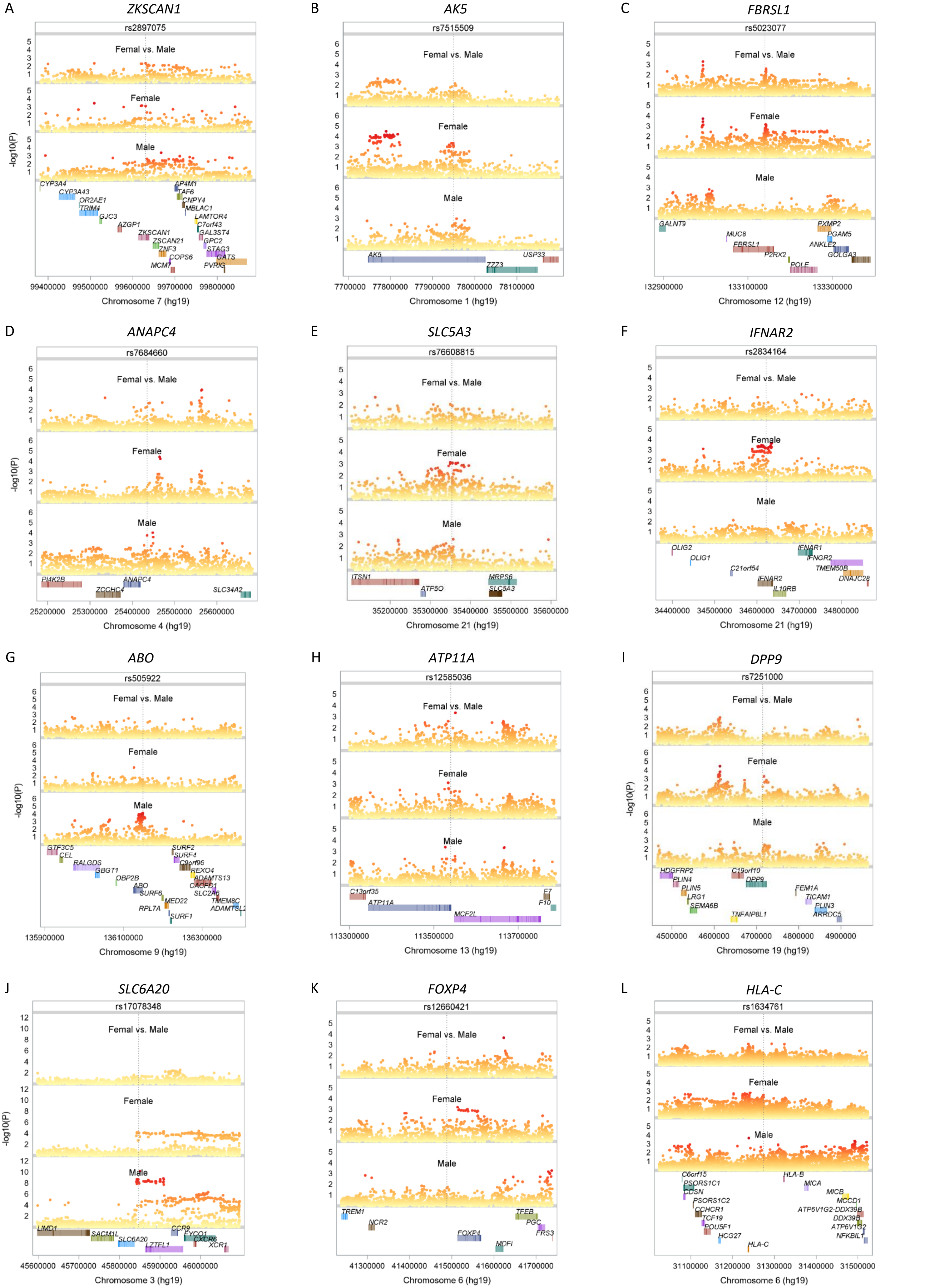
A ZKSCAN1 rs2897075
B AK5 rs7515509
C FBRSL1 rs5023077
D ANAPC4 rs7684660
E SLC5A3 rs76608815
F IFNAR2 rs2834164
G ABO rs505922
H ATP11A rs12585036
I DPP9 rs7251000
J SLC6A20 rs17078348
K FOXP4 rs12660421
L HLA-C rs1634761
Femal vs. Male
Female
Male
-log10(P)
Chromosome 7 (hg19)
Chromosome 1 (hg19)
Chromosome 12 (hg19)
Chromosome 4 (hg19)
Chromosome 21 (hg19)
Chromosome 9 (hg19)
Chromosome 13 (hg19)
Chromosome 19 (hg19)
Chromosome 3 (hg19)
Chromosome 6 (hg19)

Figure S6

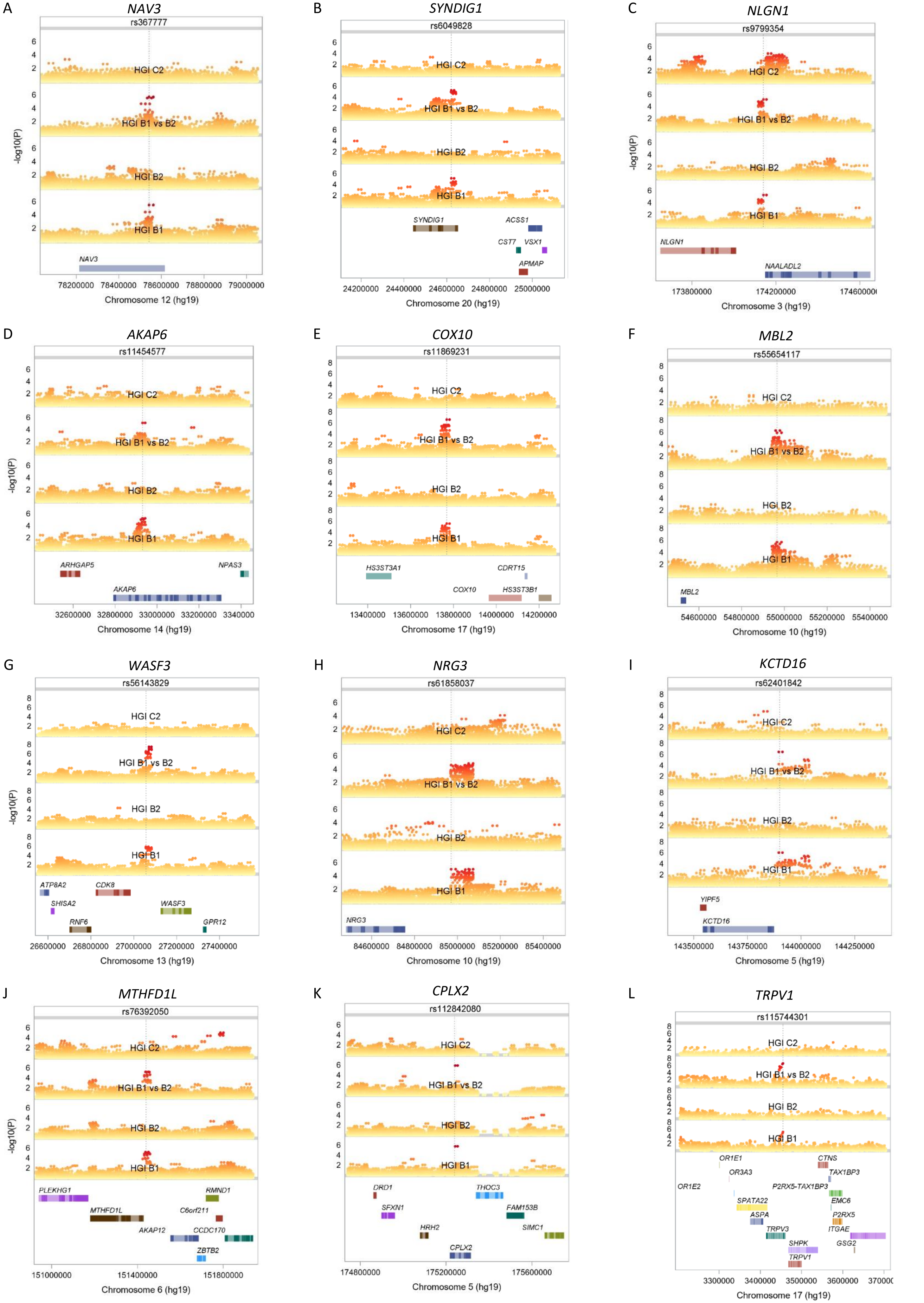

A NAV3 rs367777
HGI C2
HGI B1 vs B2
HGI B2
HGI B1
-log10(P)
NAV3
Chromosome 12 (hg19)
B SYNDIG1 rs6049828
SYNDIG1
ACSS1
CST7
VSX1
APMAP
Chromosome 20 (hg19)
C NLGN1 rs9799354
NLGN1
NAALADL2
Chromosome 3 (hg19)
D AKAP6 rs11454577
ARHGAP5
NPAS3
AKAP6
Chromosome 14 (hg19)
E COX10 rs11869231
HS3ST3A1
CDRT15
COX10
HS3ST3B1
Chromosome 17 (hg19)
F MBL2 rs55654117
MBL2
Chromosome 10 (hg19)
G WASF3 rs56143829
ATP8A2
CDK8
SHISA2
WASF3
RNF6
GPR12
Chromosome 13 (hg19)
H NRG3 rs61858037
NRG3
Chromosome 10 (hg19)
I KCTD16 rs62401842
YIPF5
KCTD16
Chromosome 5 (hg19)
J MTHFD1L rs76392050
PLEKHG1
RMND1
MTHFD1L
C6orf211
AKAP12
CCDC170
ZBTB2
Chromosome 6 (hg19)
K CPLX2 rs112842080
DRD1
THOC3
SFXN1
FAM153B
HRH2
SIMC1
CPLX2
Chromosome 5 (hg19)
L TRPV1 rs115744301
OR1E1
CTNS
OR3A3
TAX1BP3
OR1E2
P2RX5-TAX1BP3
SPATA22
EMC6
ASPA
P2RX5
TRPV3
ITGAE
SHPK
GSG2
TRPV1
Chromosome 17 (hg19)

Figure S7

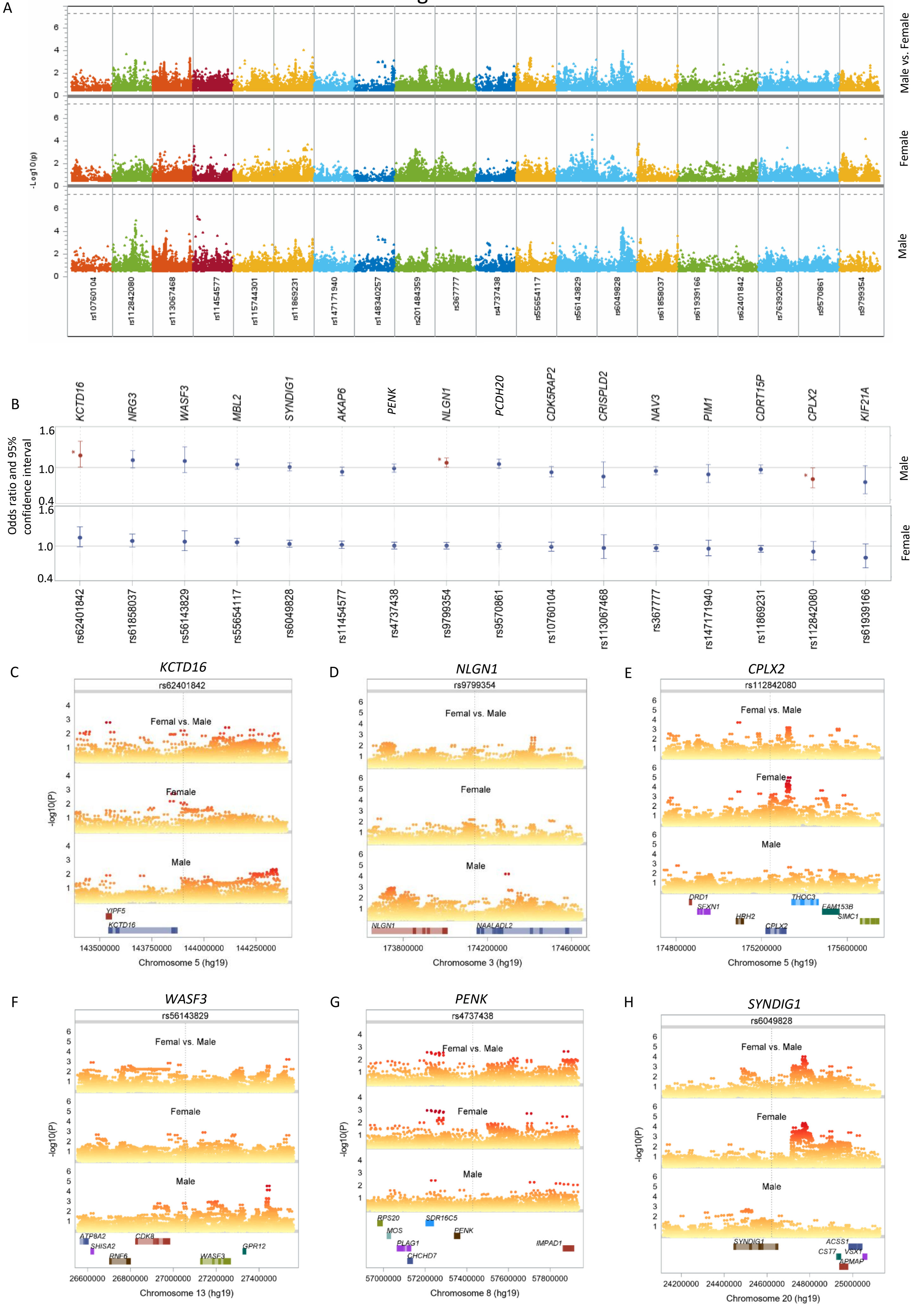
A
Male vs. Female
Female
Male
-Log10(p)
rs10760104
rs112842080
rs113067468
rs11454577
rs115744301
rs11869231
rs147171940
rs148340257
rs201484359
rs367777
rs4737438
rs55654117
rs56143829
rs6049828
rs61858037
rs61939166
rs62401842
rs76392050
rs9570861
rs9799354
B
KCTD16
NRG3
WASF3
MBL2
SYNDIG1
AKAP6
PENK
NLGN1
PCDH20
CDK5RAP2
CRISPLD2
NAV3
PIM1
CDRT15P
CPLX2
KIF21A
Odds ratio and 95% confidence interval
Male
Female
rs62401842
rs61858037
rs56143829
rs55654117
rs6049828
rs11454577
rs4737438
rs9799354
rs9570861
rs10760104
rs113067468
rs367777
rs147171940
rs11869231
rs112842080
rs61939166
C
KCTD16
rs62401842
Femal vs. Male
Female
Male
-log10(P)
YIPF5
KCTD16
143500000
143750000
144000000
144250000
Chromosome 5 (hg19)
D
NLGN1
rs9799354
Femal vs. Male
Female
Male
NLGN1
NAALADL2
173800000
174200000
174600000
Chromosome 3 (hg19)
E
CPLX2
rs112842080
Femal vs. Male
Female
Male
DRD1
SFXN1
HRH2
THOC3
FAM153B
SIMC1
CPLX2
174800000
175200000
175600000
Chromosome 5 (hg19)
F
WASF3
rs56143829
Femal vs. Male
Female
Male
-log10(P)
ATP8A2
SHISA2
CDK8
RNF6
WASF3
GPR12
26600000
26800000
27000000
27200000
27400000
Chromosome 13 (hg19)
G
PENK
rs4737438
Femal vs. Male
Female
Male
-log10(P)
RPS20
MOS
PLAG1
CHCHD7
SDR16C5
PENK
IMPAD1
57000000
57200000
57400000
57600000
57800000
Chromosome 8 (hg19)
H
SYNDIG1
rs6049828
Femal vs. Male
Female
Male
-log10(P)
SYNDIG1
ACSS1
CST7
VSX1
APMAP
24200000
24400000
24600000
24800000
25000000
Chromosome 20 (hg19)

Figure S8

Long COVID association signals for SNPs in the SNP category "Severe COVID-19-Specific SNPs"

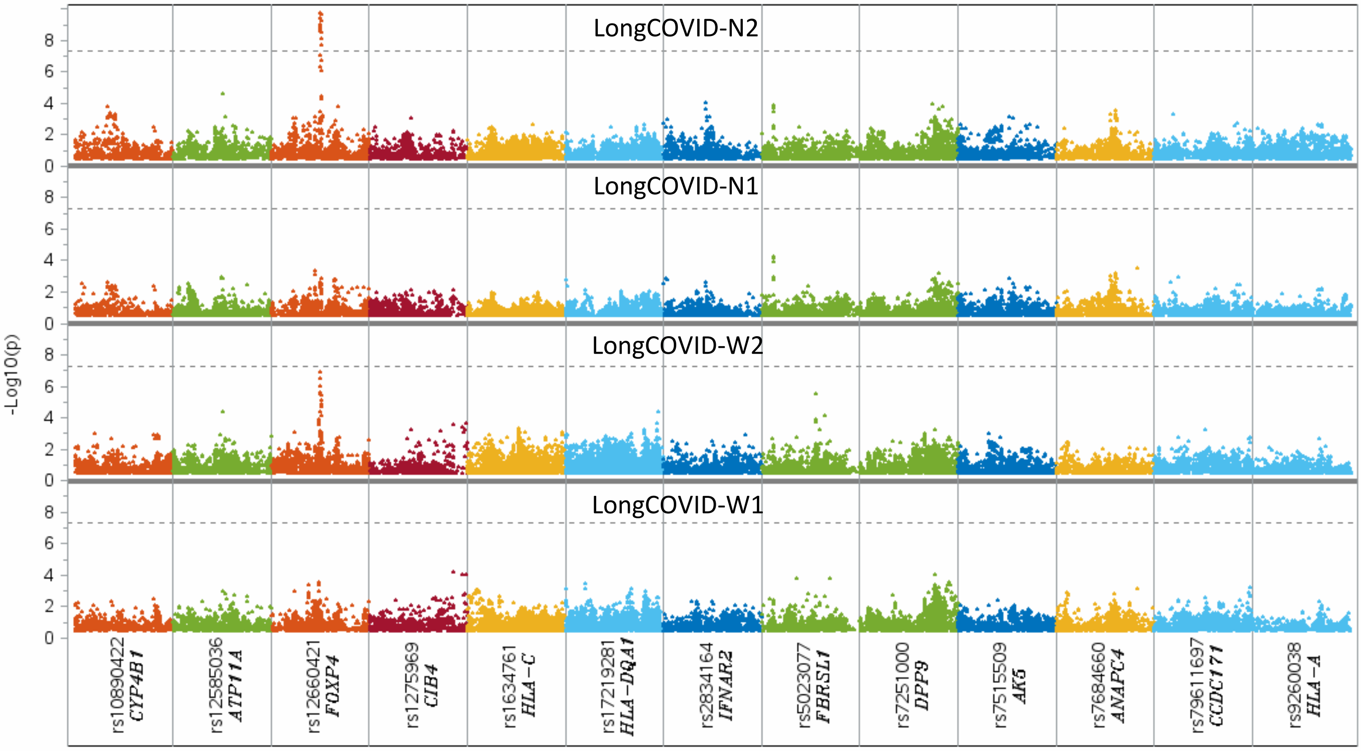

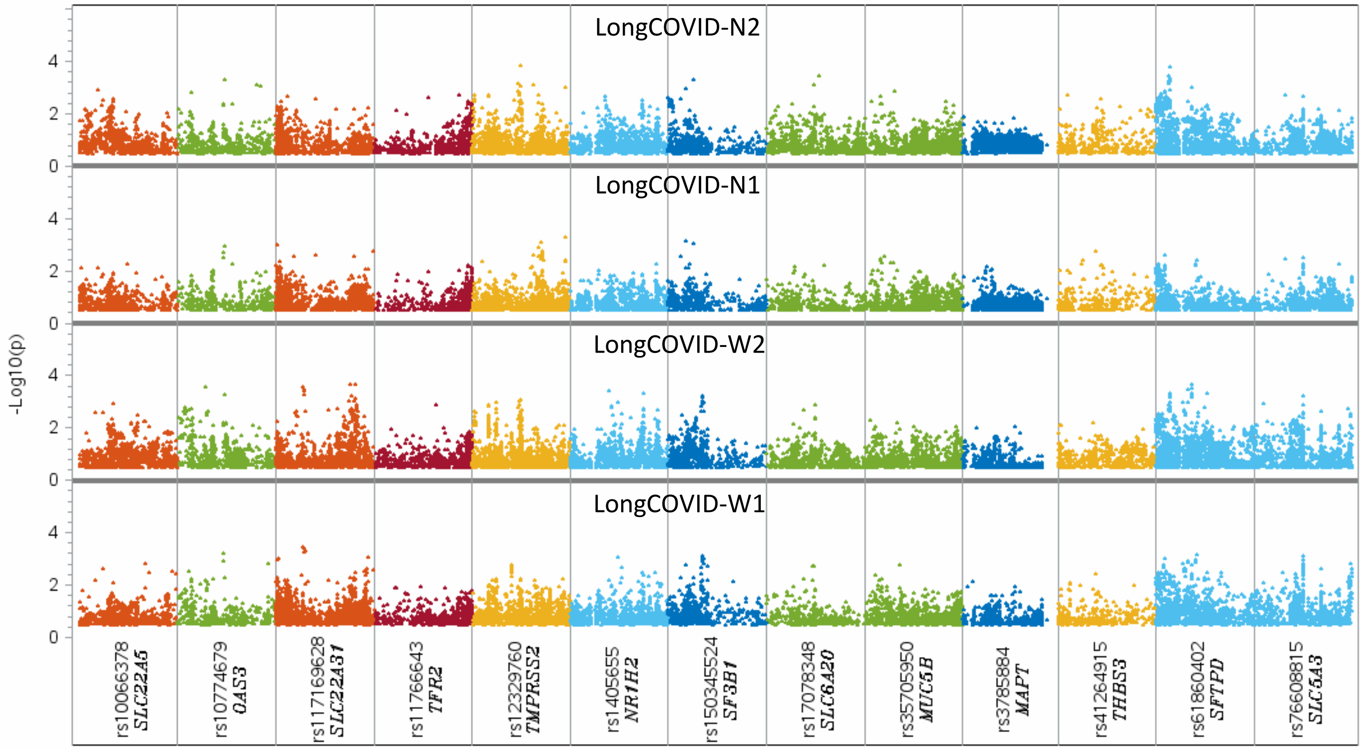

Figure S9

Long COVID association signals for SNPs in the SNP category "Mild COVID-19-Specific SNPs"

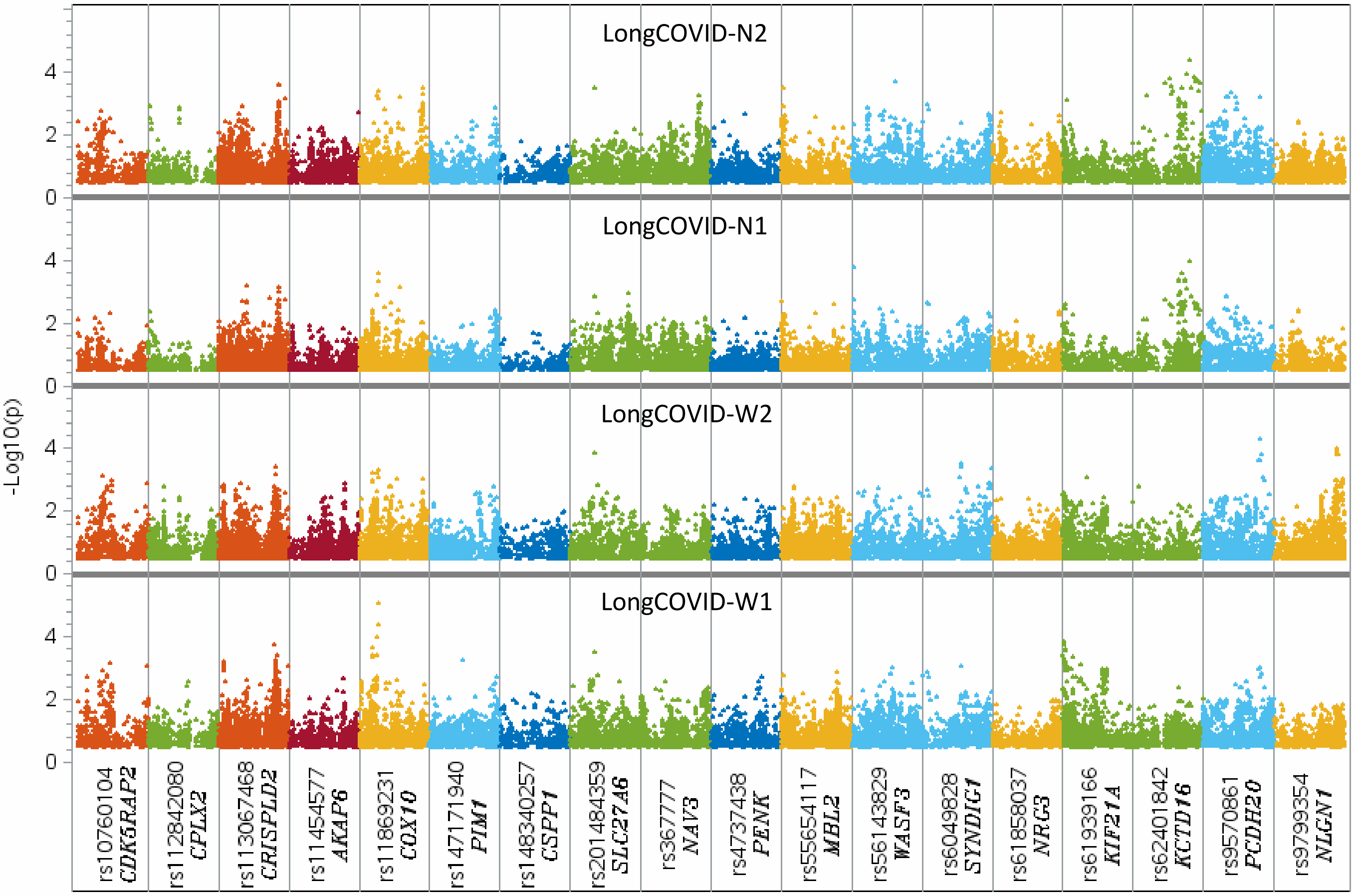

Long COVID association signals for SNPs in the SNP category "SNPs Associated with Both Severe and Mild COVID-19"

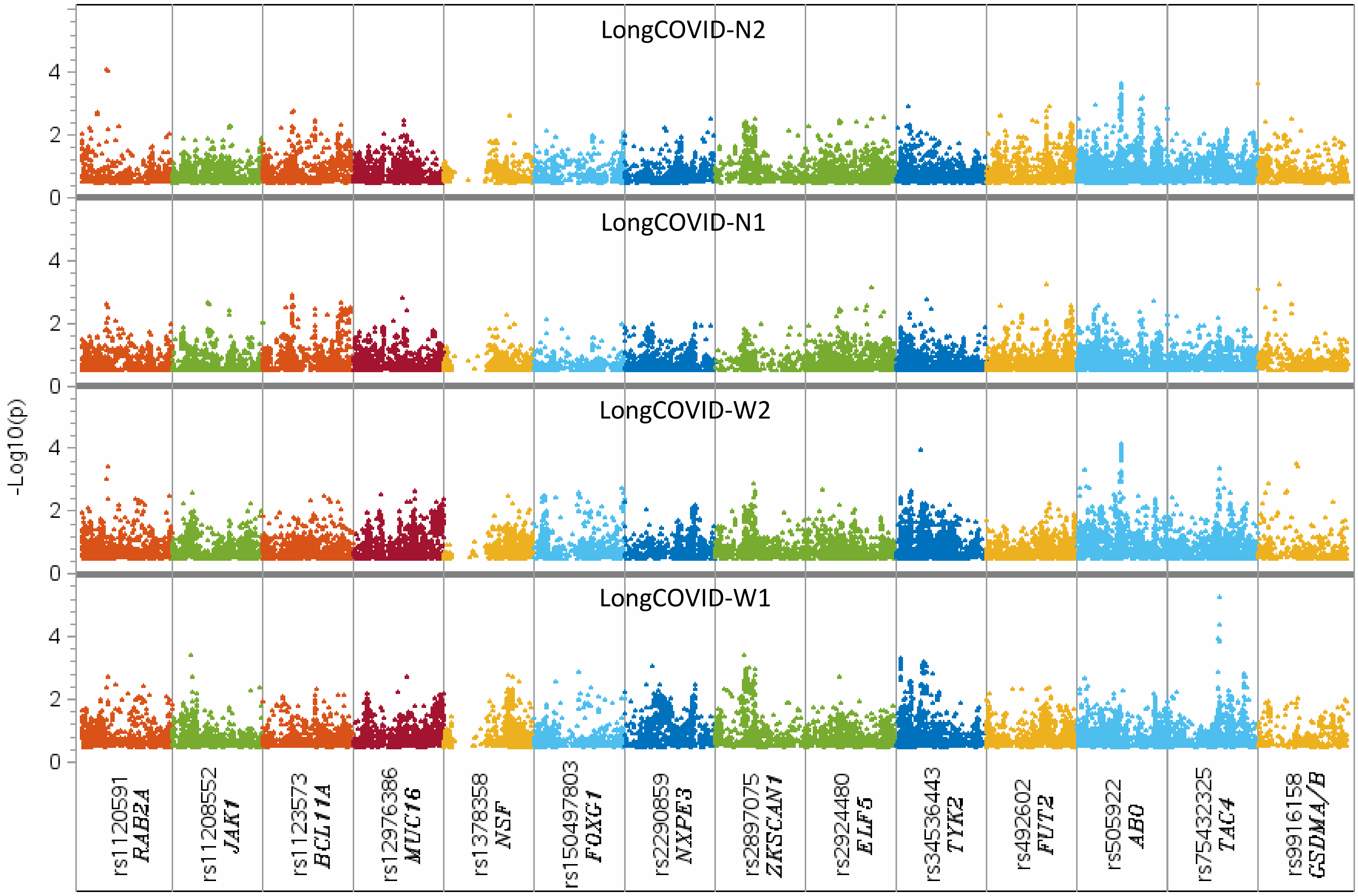

Figure S10

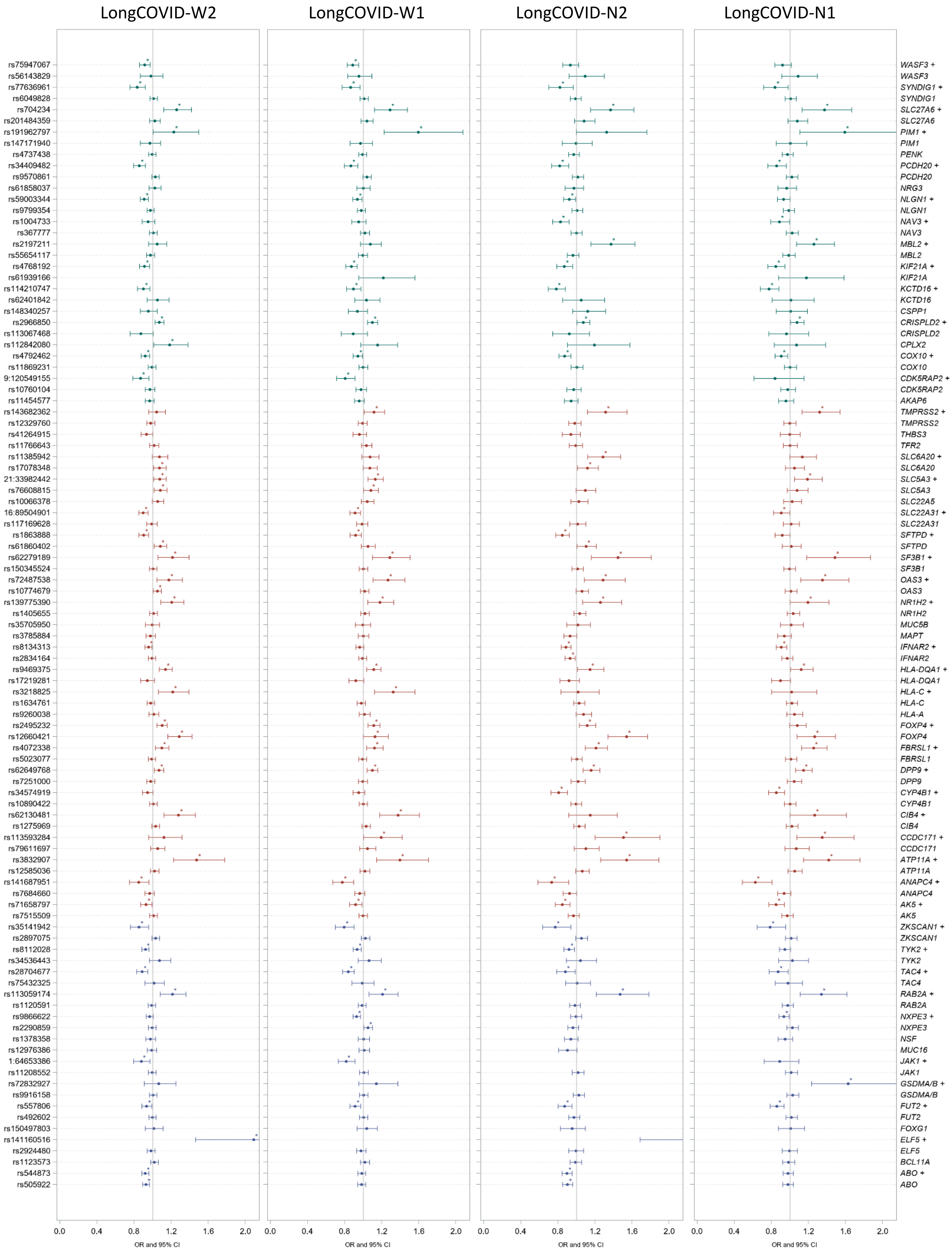
LongCOVID-W2
LongCOVID-W1
LongCOVID-N2
LongCOVID-N1
OR and 95% CI
0.0
0.4
0.8
1.2
1.6
2.0
rs75947067 WASF3 +
rs56143829 WASF3
rs77636961 SYNDIG1 +
rs6049828 SYNDIG1
rs704234 SLC27A6 +
rs201484359 SLC27A6
rs191962797 PIM1 +
rs147171940 PIM1
rs4737438 PENK
rs34409482 PCDH20 +
rs9570861 PCDH20
rs61858037 NRG3
rs59003344 NLGN1 +
rs9799354 NLGN1
rs1004733 NAV3 +
rs367777 NAV3
rs2197211 MBL2 +
rs55654117 MBL2
rs4768192 KIF21A +
rs61939166 KIF21A
rs114210747 KCTD16 +
rs62401842 KCTD16
rs148340257 CSPP1
rs2966850 CRISPLD2 +
rs113067468 CRISPLD2
rs112842080 CPLX2
rs4792462 COX10 +
rs11869231 COX10
9:120549155 CDK5RAP2 +
rs10760104 CDK5RAP2
rs11454577 AKAP6
rs143682362 TMPRSS2 +
rs12329760 TMPRSS2
rs41264915 THBS3
rs11766643 TFR2
rs11385942 SLC6A20 +
rs17078348 SLC6A20
21:33982442 SLC5A3 +
rs76608815 SLC5A3
rs10066378 SLC22A5
16:89504901 SLC22A31 +
rs117169628 SLC22A31
rs1863888 SFTPD +
rs61860402 SFTPD
rs62279189 SF3B1 +
rs150345524 SF3B1
rs72487538 OAS3 +
rs10774679 OAS3
rs139775390 NR1H2 +
rs1405655 NR1H2
rs35705950 MUC5B
rs3785884 MAPT
rs8134313 IFNAR2 +
rs2834164 IFNAR2
rs9469375 HLA-DQA1 +
rs17219281 HLA-DQA1
rs3218825 HLA-C +
rs1634761 HLA-C
rs9260038 HLA-A
rs2495232 FOXP4 +
rs12660421 FOXP4
rs4072338 FBRSL1 +
rs5023077 FBRSL1
rs62649768 DPP9 +
rs7251000 DPP9
rs34574919 CYP4B1 +
rs10890422 CYP4B1
rs62130481 CIB4 +
rs1275969 CIB4
rs113593284 CCDC171 +
rs79611697 CCDC171
rs3832907 ATP11A +
rs12585036 ATP11A
rs141687951 ANAPC4 +
rs7684660 ANAPC4
rs71658797 AK5 +
rs7515509 AK5
rs35141942 ZKSCAN1 +
rs2897075 ZKSCAN1
rs8112028 TYK2 +
rs34536443 TYK2
rs28704677 TAC4 +
rs75432325 TAC4
rs113059174 RAB2A +
rs1120591 RAB2A
rs9866622 NXPE3 +
rs2290859 NXPE3
rs1378358 NSF
rs12976386 MUC16
1:64653386 JAK1 +
rs11208552 JAK1
rs72832927 GSDMA/B +
rs9916158 GSDMA/B
rs557806 FUT2 +
rs492602 FUT2
rs150497803 FOXG1
rs141160516 ELF5 +
rs2924480 ELF5
rs1123573 BCL11A
rs544873 ABO +
rs505922 ABO

Figure S11

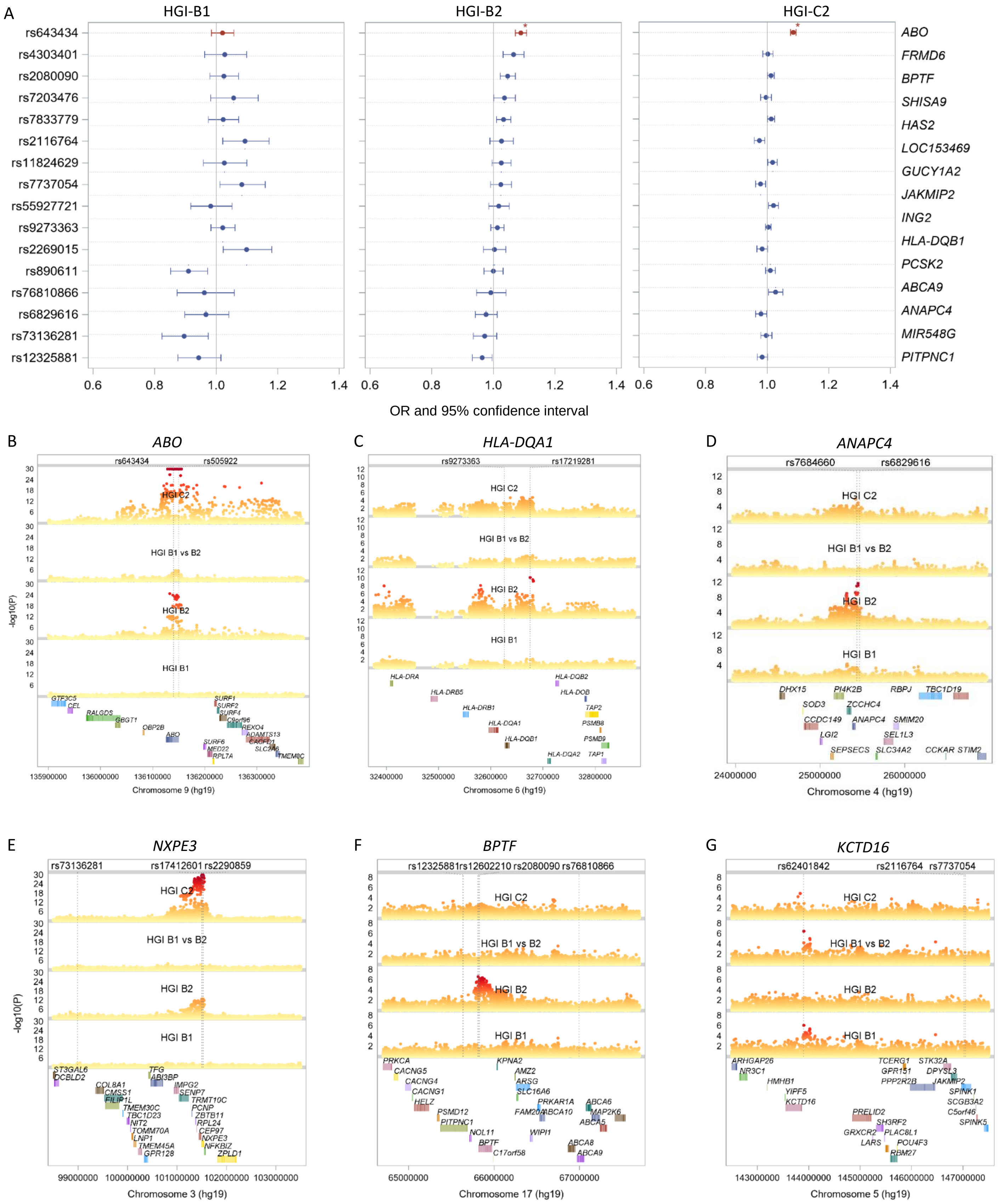
A
HGI-B1
HGI-B2
HGI-C2
rs643434
rs4303401
rs2080090
rs7203476
rs7833779
rs2116764
rs11824629
rs7737054
rs55927721
rs9273363
rs2269015
rs890611
rs76810866
rs6829616
rs73136281
rs12325881
ABO
FRMD6
BPTF
SHISA9
HAS2
LOC153469
GUCY1A2
JAKMIP2
ING2
HLA-DQB1
PCSK2
ABCA9
ANAPC4
MIR548G
PITPNC1
0.6
0.8
1.0
1.2
1.4
OR and 95% confidence interval
B
ABO
rs643434
rs505922
HGI C2
HGI B1 vs B2
HGI B2
HGI B1
-log10(P)
Chromosome 9 (hg19)
C
HLA-DQA1
rs9273363
rs17219281
Chromosome 6 (hg19)
D
ANAPC4
rs7684660
rs6829616
Chromosome 4 (hg19)
E
NXPE3
rs73136281
rs17412601 rs2290859
Chromosome 3 (hg19)
F
BPTF
rs12325881 rs12602210 rs2080090 rs76810866
Chromosome 17 (hg19)
G
KCTD16
rs62401842
rs2116764 rs7737054
Chromosome 5 (hg19)

Figure S12

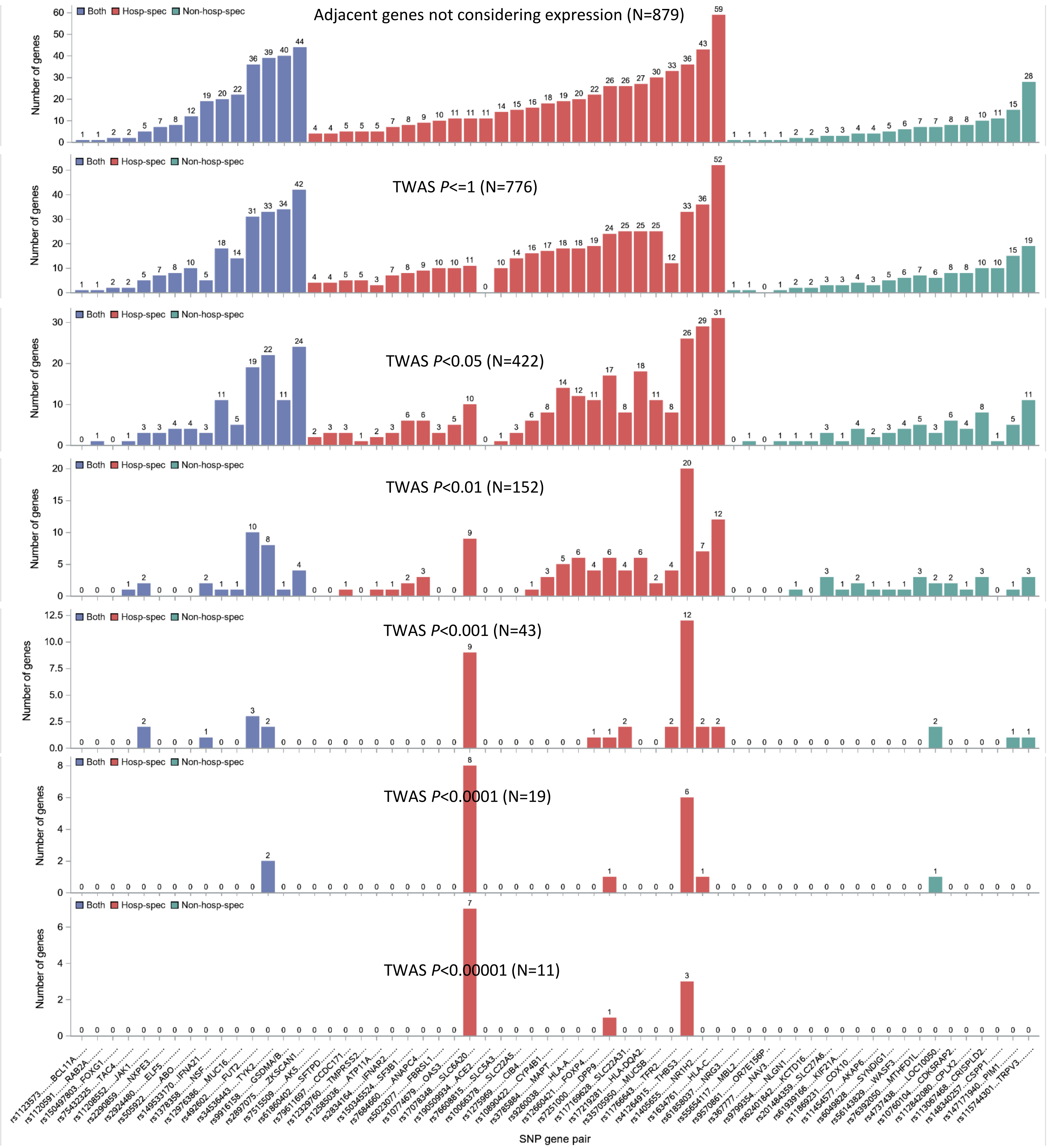

Adjacent genes not considering expression (N=879)
TWAS P<=1 (N=776)
TWAS P<0.05 (N=422)
TWAS P<0.01 (N=152)
TWAS P<0.001 (N=43)
TWAS P<0.0001 (N=19)
TWAS P<0.00001 (N=11)
Both
Hosp-spec
Non-hosp-spec
Number of genes
SNP gene pair

Figure S13

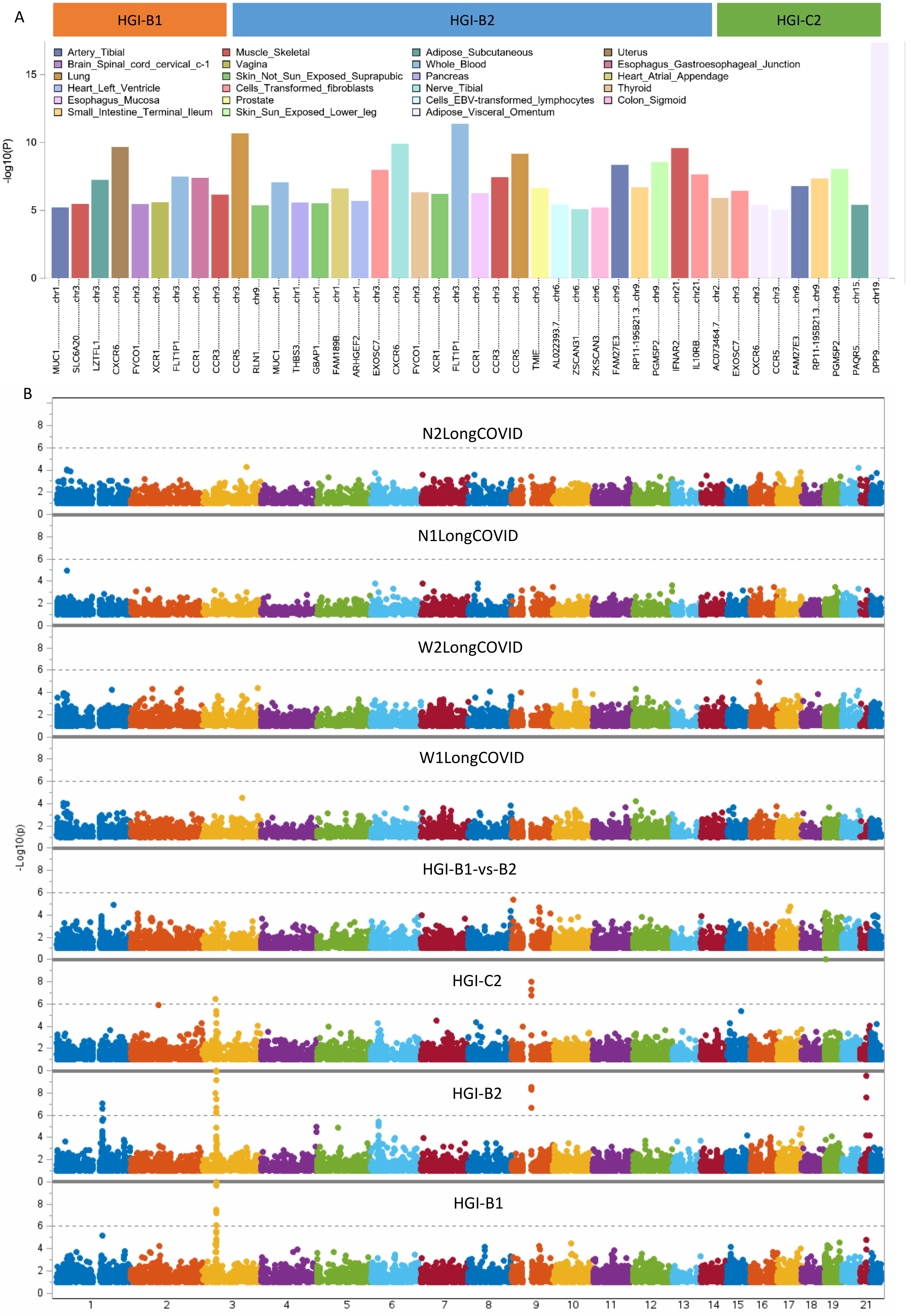

A
HGI-B1
HGI-B2
HGI-C2
Artery_Tibial
Brain_Spinal_cord_cervical_c-1
Lung
Heart_Left_Ventricle
Esophagus_Mucosa
Small_Intestine_Terminal_Ileum
Muscle_Skeletal
Vagina
Skin_Not_Sun_Exposed_Suprapubic
Cells_Transformed_fibroblasts
Prostate
Skin_Sun_Exposed_Lower_leg
Adipose_Subcutaneous
Whole_Blood
Pancreas
Nerve_Tibial
Cells_EBV-transformed_lymphocytes
Adipose_Visceral_Omentum
Uterus
Esophagus_Gastroesophageal_Junction
Heart_Atrial_Appendage
Thyroid
Colon_Sigmoid
-log10(P)
MUC1 chr1
SLC6A20 chr3
LZTFL1 chr3
CXCR6 chr3
FYCO1 chr3
XCR1 chr3
FLT1P1 chr3
CCR1 chr3
CCR3 chr3
CCR5 chr3
RLN1 chr9
MUC1 chr1
THBS3 chr1
GBAP1 chr1
FAM189B chr1
ARHGEF2 chr1
EXOSC7 chr3
CXCR6 chr3
FYCO1 chr3
XCR1 chr3
FLT1P1 chr3
CCR1 chr3
CCR3 chr3
CCR5 chr3
TMIE chr3
AL022393.7 chr6
ZSCAN31 chr6
ZKSCAN3 chr6
FAM27E3 chr9
RP11-195B21.3 chr9
PGM5P2 chr9
IFNAR2 chr21
IL10RB chr21
AC073464.7 chr2
EXOSC7 chr3
CXCR6 chr3
CCR5 chr3
FAM27E3 chr9
RP11-195B21.3 chr9
PGM5P2 chr9
PAQR5 chr15
DPP9 chr19
B
N2LongCOVID
N1LongCOVID
W2LongCOVID
W1LongCOVID
HGI-B1-vs-B2
HGI-C2
HGI-B2
HGI-B1
-Log10(p)
1 2 3 4 5 6 7 8 9 10 11 12 13 14 15 16 17 18 19 21

Figure S14

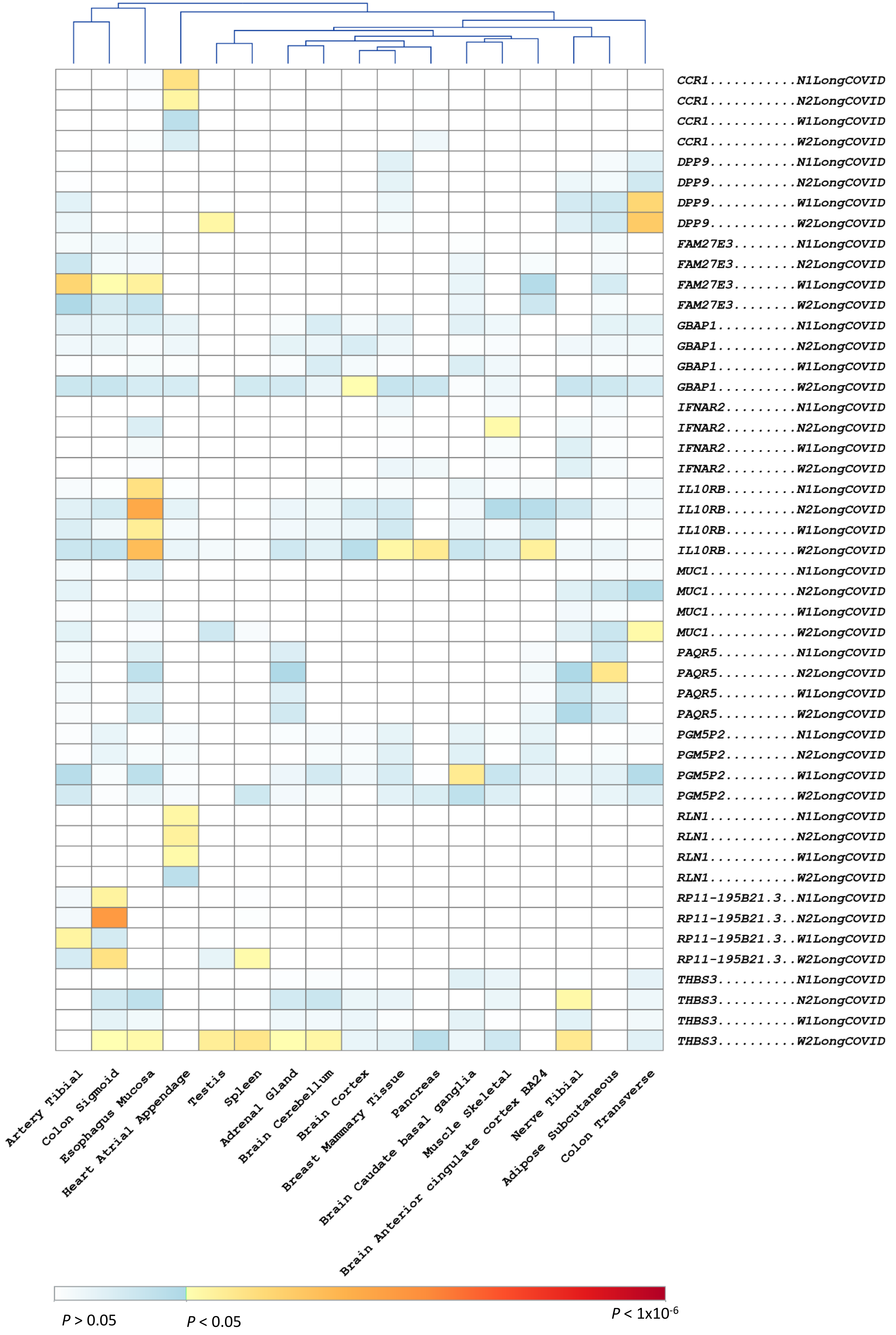

CCR1...........N1LongCOVID
CCR1...........N2LongCOVID
CCR1...........W1LongCOVID
CCR1...........W2LongCOVID
DPP9...........N1LongCOVID
DPP9...........N2LongCOVID
DPP9...........W1LongCOVID
DPP9...........W2LongCOVID
FAM27E3........N1LongCOVID
FAM27E3........N2LongCOVID
FAM27E3........W1LongCOVID
FAM27E3........W2LongCOVID
GBAP1..........N1LongCOVID
GBAP1..........N2LongCOVID
GBAP1..........W1LongCOVID
GBAP1..........W2LongCOVID
IFNAR2.........N1LongCOVID
IFNAR2.........N2LongCOVID
IFNAR2.........W1LongCOVID
IFNAR2.........W2LongCOVID
IL10RB.........N1LongCOVID
IL10RB.........N2LongCOVID
IL10RB.........W1LongCOVID
IL10RB.........W2LongCOVID
MUC1...........N1LongCOVID
MUC1...........N2LongCOVID
MUC1...........W1LongCOVID
MUC1...........W2LongCOVID
PAQR5..........N1LongCOVID
PAQR5..........N2LongCOVID
PAQR5..........W1LongCOVID
PAQR5..........W2LongCOVID
PGM5P2.........N1LongCOVID
PGM5P2.........N2LongCOVID
PGM5P2.........W1LongCOVID
PGM5P2.........W2LongCOVID
RLN1...........N1LongCOVID
RLN1...........N2LongCOVID
RLN1...........W1LongCOVID
RLN1...........W2LongCOVID
RP11-195B21.3..N1LongCOVID
RP11-195B21.3..N2LongCOVID
RP11-195B21.3..W1LongCOVID
RP11-195B21.3..W2LongCOVID
THBS3..........N1LongCOVID
THBS3..........N2LongCOVID
THBS3..........W1LongCOVID
THBS3..........W2LongCOVID
Artery Tibial
Colon Sigmoid
Esophagus Mucosa
Heart Atrial Appendage
Testis
Spleen
Adrenal Gland
Brain Cerebellum
Brain Cortex
Breast Mammary Tissue
Pancreas
Brain Caudate basal ganglia
Muscle Skeletal
Brain Anterior cingulate cortex BA24
Nerve Tibial
Adipose Subcutaneous
Colon Transverse
P > 0.05
P < 0.05
P < 1x10-6

Figure S15

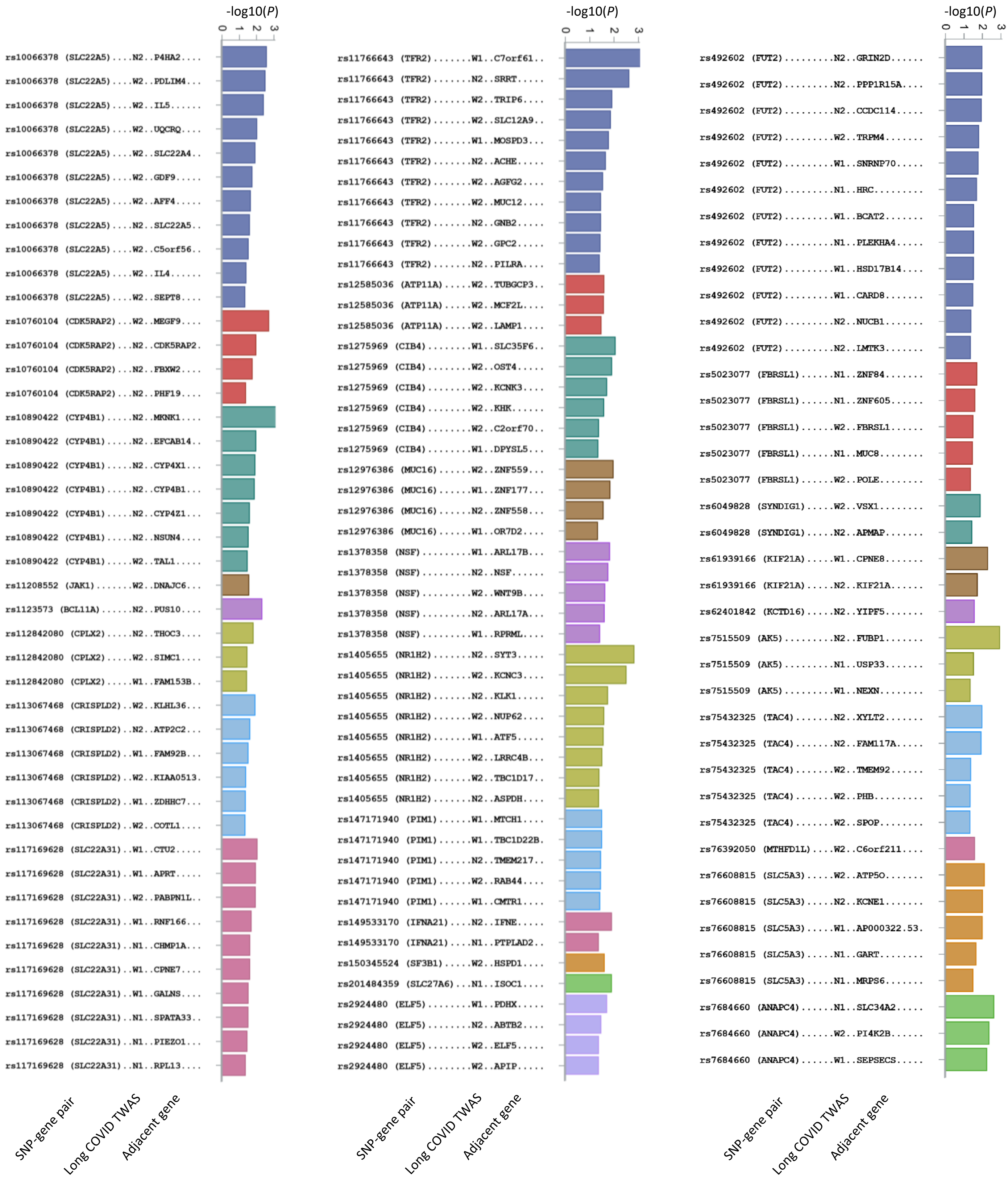

Figure S16

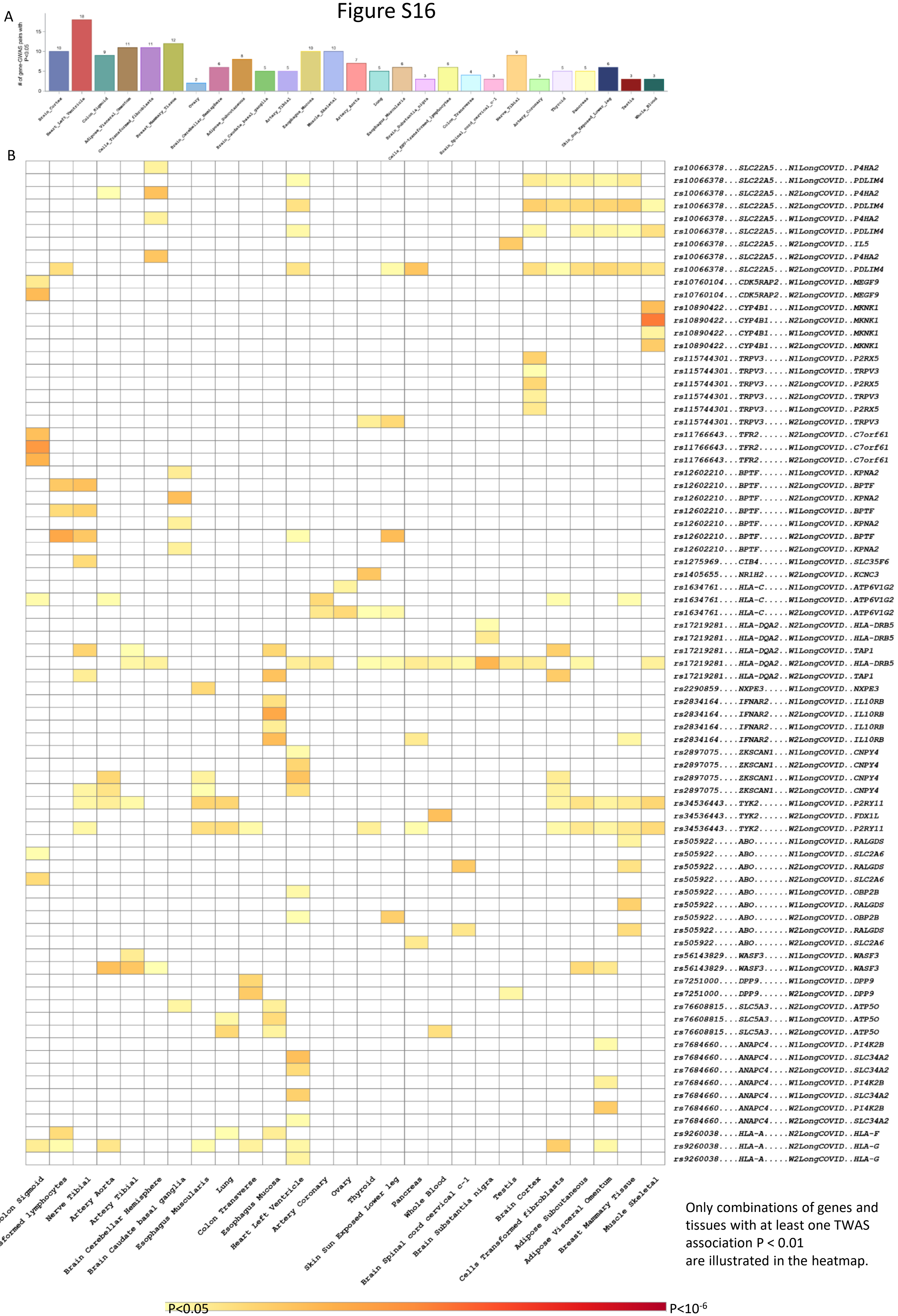

Only combinations of genes and tissues with at least one TWAS association P < 0.01 are illustrated in the heatmap.

Figure S17

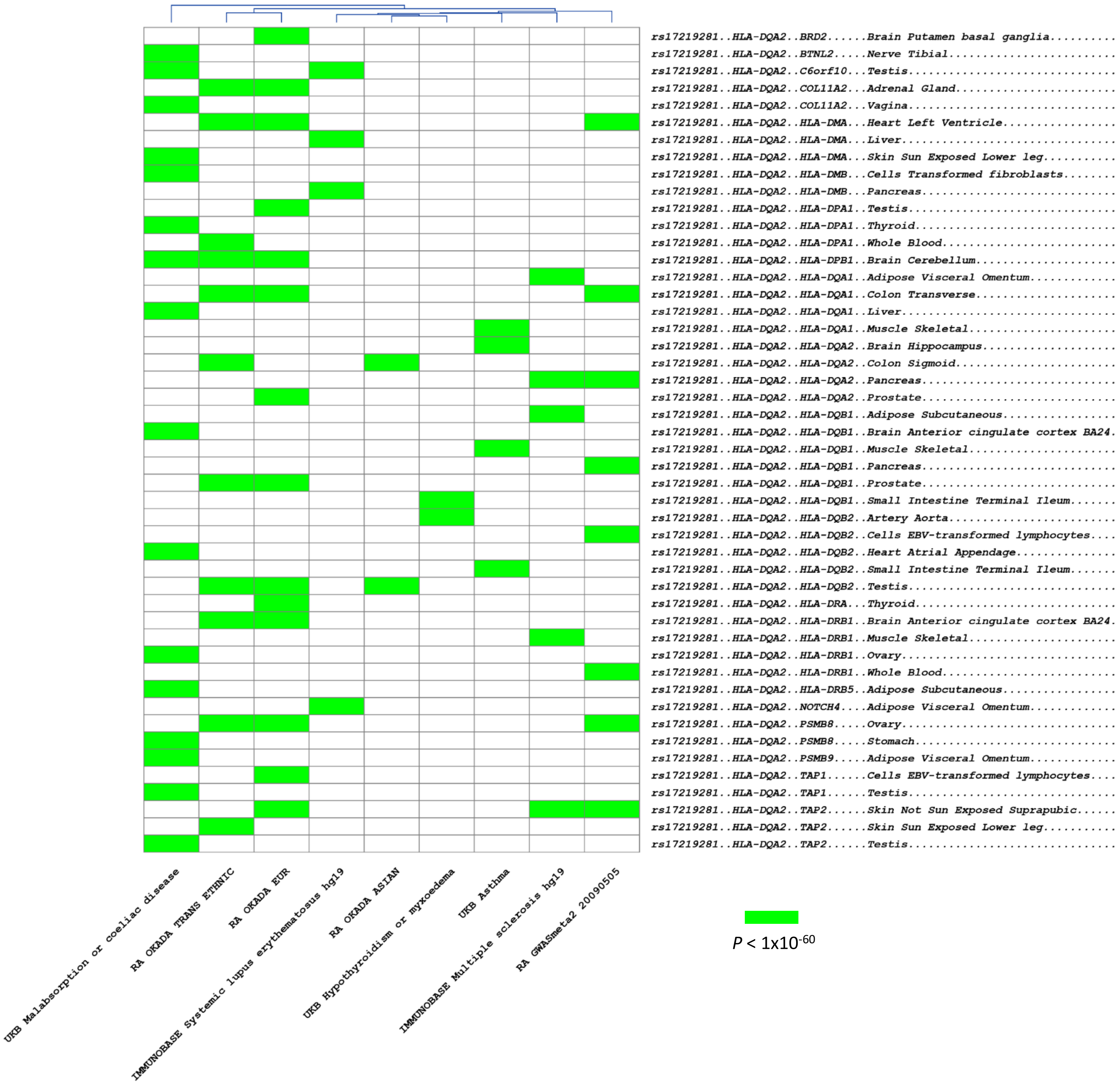

rs17219281..HLA-DQA2..BRD2......Brain Putamen basal ganglia..........
rs17219281..HLA-DQA2..BTNL2.....Nerve Tibial........................
rs17219281..HLA-DQA2..C6orf10...Testis..............................
rs17219281..HLA-DQA2..COL11A2...Adrenal Gland.......................
rs17219281..HLA-DQA2..COL11A2...Vagina..............................
rs17219281..HLA-DQA2..HLA-DMA...Heart Left Ventricle................
rs17219281..HLA-DQA2..HLA-DMA...Liver...............................
rs17219281..HLA-DQA2..HLA-DMA...Skin Sun Exposed Lower leg..........
rs17219281..HLA-DQA2..HLA-DMB...Cells Transformed fibroblasts.......
rs17219281..HLA-DQA2..HLA-DMB...Pancreas............................
rs17219281..HLA-DQA2..HLA-DPA1..Testis..............................
rs17219281..HLA-DQA2..HLA-DPA1..Thyroid.............................
rs17219281..HLA-DQA2..HLA-DPA1..Whole Blood.........................
rs17219281..HLA-DQA2..HLA-DPB1..Brain Cerebellum....................
rs17219281..HLA-DQA2..HLA-DQA1..Adipose Visceral Omentum............
rs17219281..HLA-DQA2..HLA-DQA1..Colon Transverse....................
rs17219281..HLA-DQA2..HLA-DQA1..Liver...............................
rs17219281..HLA-DQA2..HLA-DQA1..Muscle Skeletal.....................
rs17219281..HLA-DQA2..HLA-DQA2..Brain Hippocampus...................
rs17219281..HLA-DQA2..HLA-DQA2..Colon Sigmoid.......................
rs17219281..HLA-DQA2..HLA-DQA2..Pancreas............................
rs17219281..HLA-DQA2..HLA-DQA2..Prostate............................
rs17219281..HLA-DQA2..HLA-DQB1..Adipose Subcutaneous................
rs17219281..HLA-DQA2..HLA-DQB1..Brain Anterior cingulate cortex BA24.
rs17219281..HLA-DQA2..HLA-DQB1..Muscle Skeletal.....................
rs17219281..HLA-DQA2..HLA-DQB1..Pancreas............................
rs17219281..HLA-DQA2..HLA-DQB1..Prostate............................
rs17219281..HLA-DQA2..HLA-DQB1..Small Intestine Terminal Ileum......
rs17219281..HLA-DQA2..HLA-DQB2..Artery Aorta........................
rs17219281..HLA-DQA2..HLA-DQB2..Cells EBV-transformed lymphocytes....
rs17219281..HLA-DQA2..HLA-DQB2..Heart Atrial Appendage..............
rs17219281..HLA-DQA2..HLA-DQB2..Small Intestine Terminal Ileum......
rs17219281..HLA-DQA2..HLA-DQB2..Testis..............................
rs17219281..HLA-DQA2..HLA-DRA...Thyroid.............................
rs17219281..HLA-DQA2..HLA-DRB1..Brain Anterior cingulate cortex BA24.
rs17219281..HLA-DQA2..HLA-DRB1..Muscle Skeletal.....................
rs17219281..HLA-DQA2..HLA-DRB1..Ovary...............................
rs17219281..HLA-DQA2..HLA-DRB1..Whole Blood.........................
rs17219281..HLA-DQA2..HLA-DRB5..Adipose Subcutaneous................
rs17219281..HLA-DQA2..NOTCH4....Adipose Visceral Omentum............
rs17219281..HLA-DQA2..PSMB8.....Ovary...............................
rs17219281..HLA-DQA2..PSMB8.....Stomach.............................
rs17219281..HLA-DQA2..PSMB9.....Adipose Visceral Omentum............
rs17219281..HLA-DQA2..TAP1......Cells EBV-transformed lymphocytes....
rs17219281..HLA-DQA2..TAP1......Testis..............................
rs17219281..HLA-DQA2..TAP2......Skin Not Sun Exposed Suprapubic......
rs17219281..HLA-DQA2..TAP2......Skin Sun Exposed Lower leg..........
rs17219281..HLA-DQA2..TAP2......Testis..............................
UKB Malabsorption or coeliac disease
RA OKADA TRANS ETHNIC
RA OKADA EUR
IMMUNOBASE Systemic lupus erythematosus hg19
RA OKADA ASIAN
UKB Hypothyroidism or myxoedema
UKB Asthma
IMMUNOBASE Multiple sclerosis hg19
RA GWASmeta2 20090505
P < 1x10-60

Figure S18

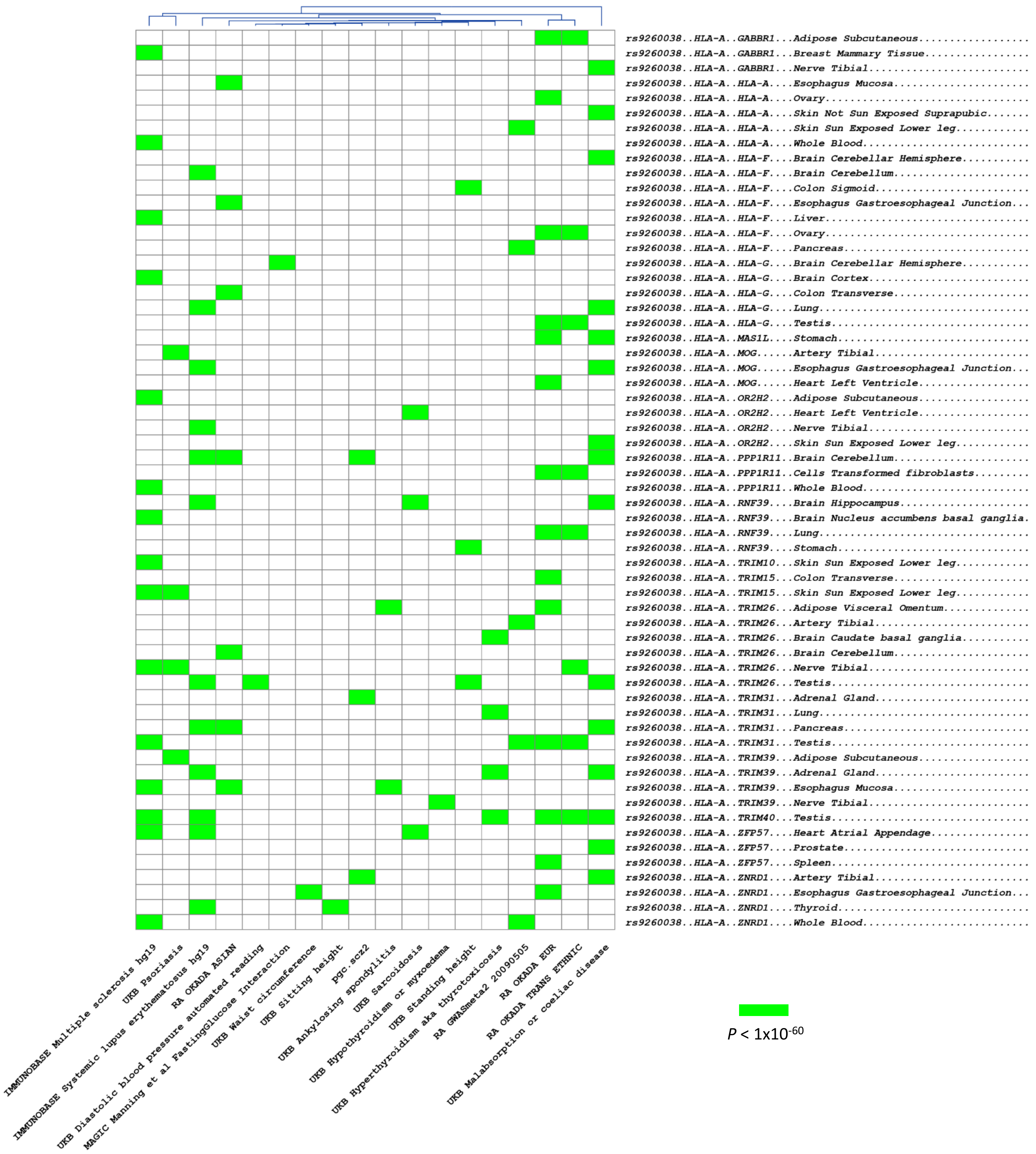

rs9260038..HLA-A..GABBR1...Adipose Subcutaneous
rs9260038..HLA-A..GABBR1...Breast Mammary Tissue
rs9260038..HLA-A..GABBR1...Nerve Tibial
rs9260038..HLA-A..HLA-A....Esophagus Mucosa
rs9260038..HLA-A..HLA-A....Ovary
rs9260038..HLA-A..HLA-A....Skin Not Sun Exposed Suprapubic
rs9260038..HLA-A..HLA-A....Skin Sun Exposed Lower leg
rs9260038..HLA-A..HLA-A....Whole Blood
rs9260038..HLA-A..HLA-F....Brain Cerebellar Hemisphere
rs9260038..HLA-A..HLA-F....Brain Cerebellum
rs9260038..HLA-A..HLA-F....Colon Sigmoid
rs9260038..HLA-A..HLA-F....Esophagus Gastroesophageal Junction
rs9260038..HLA-A..HLA-F....Liver
rs9260038..HLA-A..HLA-F....Ovary
rs9260038..HLA-A..HLA-F....Pancreas
rs9260038..HLA-A..HLA-G....Brain Cerebellar Hemisphere
rs9260038..HLA-A..HLA-G....Brain Cortex
rs9260038..HLA-A..HLA-G....Colon Transverse
rs9260038..HLA-A..HLA-G....Lung
rs9260038..HLA-A..HLA-G....Testis
rs9260038..HLA-A..MAS1L....Stomach
rs9260038..HLA-A..MOG......Artery Tibial
rs9260038..HLA-A..MOG......Esophagus Gastroesophageal Junction
rs9260038..HLA-A..MOG......Heart Left Ventricle
rs9260038..HLA-A..OR2H2....Adipose Subcutaneous
rs9260038..HLA-A..OR2H2....Heart Left Ventricle
rs9260038..HLA-A..OR2H2....Nerve Tibial
rs9260038..HLA-A..OR2H2....Skin Sun Exposed Lower leg
rs9260038..HLA-A..PPP1R11..Brain Cerebellum
rs9260038..HLA-A..PPP1R11..Cells Transformed fibroblasts
rs9260038..HLA-A..PPP1R11..Whole Blood
rs9260038..HLA-A..RNF39....Brain Hippocampus
rs9260038..HLA-A..RNF39....Brain Nucleus accumbens basal ganglia
rs9260038..HLA-A..RNF39....Lung
rs9260038..HLA-A..RNF39....Stomach
rs9260038..HLA-A..TRIM10...Skin Sun Exposed Lower leg
rs9260038..HLA-A..TRIM15...Colon Transverse
rs9260038..HLA-A..TRIM15...Skin Sun Exposed Lower leg
rs9260038..HLA-A..TRIM26...Adipose Visceral Omentum
rs9260038..HLA-A..TRIM26...Artery Tibial
rs9260038..HLA-A..TRIM26...Brain Caudate basal ganglia
rs9260038..HLA-A..TRIM26...Brain Cerebellum
rs9260038..HLA-A..TRIM26...Nerve Tibial
rs9260038..HLA-A..TRIM26...Testis
rs9260038..HLA-A..TRIM31...Adrenal Gland
rs9260038..HLA-A..TRIM31...Lung
rs9260038..HLA-A..TRIM31...Pancreas
rs9260038..HLA-A..TRIM31...Testis
rs9260038..HLA-A..TRIM39...Adipose Subcutaneous
rs9260038..HLA-A..TRIM39...Adrenal Gland
rs9260038..HLA-A..TRIM39...Esophagus Mucosa
rs9260038..HLA-A..TRIM39...Nerve Tibial
rs9260038..HLA-A..TRIM40...Testis
rs9260038..HLA-A..ZFP57....Heart Atrial Appendage
rs9260038..HLA-A..ZFP57....Prostate
rs9260038..HLA-A..ZFP57....Spleen
rs9260038..HLA-A..ZNRD1....Artery Tibial
rs9260038..HLA-A..ZNRD1....Esophagus Gastroesophageal Junction
rs9260038..HLA-A..ZNRD1....Thyroid
rs9260038..HLA-A..ZNRD1....Whole Blood
IMMUNOBASE Multiple sclerosis hg19
UKB Psoriasis
IMMUNOBASE Systemic lupus erythematosus hg19
RA OKADA ASIAN
UKB Diastolic blood pressure automated reading
MAGIC Manning et al FastingGlucose Interaction
UKB Waist circumference
UKB Sitting height
pgc.scz2
UKB Ankylosing spondylitis
UKB Sarcoidosis
UKB Hypothyroidism or myxoedema
UKB Standing height
UKB Hyperthyroidism aka thyrotoxicosis
RA GWASmeta2 20090505
RA OKADA EUR
RA OKADA TRANS ETHNIC
UKB Malabsorption or coeliac disease
$P < 1x10^{-60}$

Figure S19

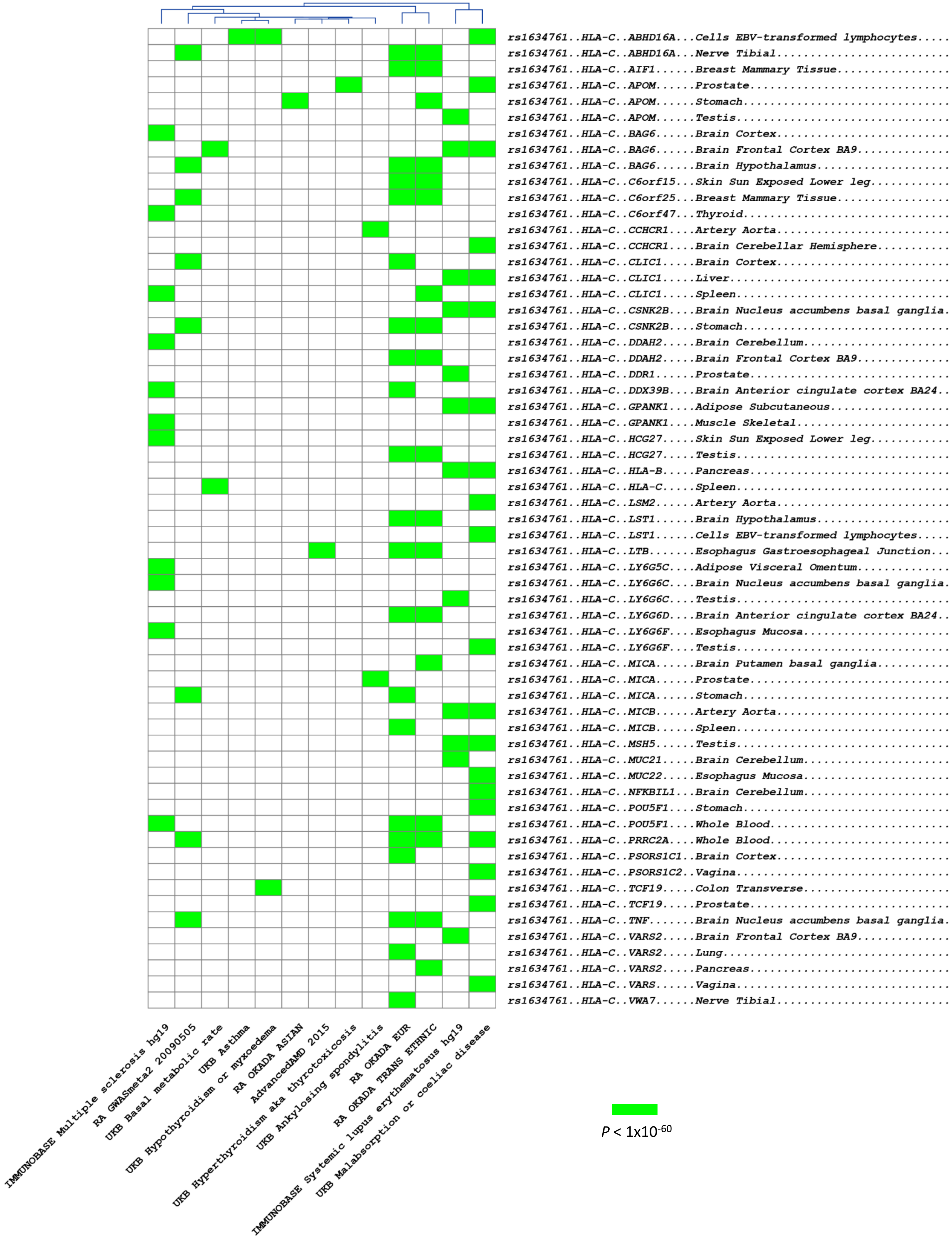
rs1634761..HLA-C..ABHD16A...Cells EBV-transformed lymphocytes.....
rs1634761..HLA-C..ABHD16A...Nerve Tibial..........................
rs1634761..HLA-C..AIF1......Breast Mammary Tissue.................
rs1634761..HLA-C..APOM......Prostate..............................
rs1634761..HLA-C..APOM......Stomach...............................
rs1634761..HLA-C..APOM......Testis................................
rs1634761..HLA-C..BAG6......Brain Cortex..........................
rs1634761..HLA-C..BAG6......Brain Frontal Cortex BA9..............
rs1634761..HLA-C..BAG6......Brain Hypothalamus....................
rs1634761..HLA-C..C6orf15...Skin Sun Exposed Lower leg............
rs1634761..HLA-C..C6orf25...Breast Mammary Tissue.................
rs1634761..HLA-C..C6orf47...Thyroid...............................
rs1634761..HLA-C..CCHCR1....Artery Aorta..........................
rs1634761..HLA-C..CCHCR1....Brain Cerebellar Hemisphere...........
rs1634761..HLA-C..CLIC1.....Brain Cortex..........................
rs1634761..HLA-C..CLIC1.....Liver.................................
rs1634761..HLA-C..CLIC1.....Spleen................................
rs1634761..HLA-C..CSNK2B....Brain Nucleus accumbens basal ganglia.
rs1634761..HLA-C..CSNK2B....Stomach...............................
rs1634761..HLA-C..DDAH2.....Brain Cerebellum......................
rs1634761..HLA-C..DDAH2.....Brain Frontal Cortex BA9..............
rs1634761..HLA-C..DDR1......Prostate..............................
rs1634761..HLA-C..DDX39B....Brain Anterior cingulate cortex BA24..
rs1634761..HLA-C..GPANK1....Adipose Subcutaneous..................
rs1634761..HLA-C..GPANK1....Muscle Skeletal.......................
rs1634761..HLA-C..HCG27.....Skin Sun Exposed Lower leg............
rs1634761..HLA-C..HCG27.....Testis................................
rs1634761..HLA-C..HLA-B.....Pancreas..............................
rs1634761..HLA-C..HLA-C.....Spleen................................
rs1634761..HLA-C..LSM2......Artery Aorta..........................
rs1634761..HLA-C..LST1......Brain Hypothalamus....................
rs1634761..HLA-C..LST1......Cells EBV-transformed lymphocytes.....
rs1634761..HLA-C..LTB.......Esophagus Gastroesophageal Junction...
rs1634761..HLA-C..LY6G5C....Adipose Visceral Omentum..............
rs1634761..HLA-C..LY6G6C....Brain Nucleus accumbens basal ganglia.
rs1634761..HLA-C..LY6G6C....Testis................................
rs1634761..HLA-C..LY6G6D....Brain Anterior cingulate cortex BA24..
rs1634761..HLA-C..LY6G6F....Esophagus Mucosa......................
rs1634761..HLA-C..LY6G6F....Testis................................
rs1634761..HLA-C..MICA......Brain Putamen basal ganglia...........
rs1634761..HLA-C..MICA......Prostate..............................
rs1634761..HLA-C..MICA......Stomach...............................
rs1634761..HLA-C..MICB......Artery Aorta..........................
rs1634761..HLA-C..MICB......Spleen................................
rs1634761..HLA-C..MSH5......Testis................................
rs1634761..HLA-C..MUC21.....Brain Cerebellum......................
rs1634761..HLA-C..MUC22.....Esophagus Mucosa......................
rs1634761..HLA-C..NFKBIL1...Brain Cerebellum......................
rs1634761..HLA-C..POU5F1....Stomach...............................
rs1634761..HLA-C..POU5F1....Whole Blood...........................
rs1634761..HLA-C..PRRC2A....Whole Blood...........................
rs1634761..HLA-C..PSORS1C1..Brain Cortex..........................
rs1634761..HLA-C..PSORS1C2..Vagina................................
rs1634761..HLA-C..TCF19.....Colon Transverse......................
rs1634761..HLA-C..TCF19.....Prostate..............................
rs1634761..HLA-C..TNF.......Brain Nucleus accumbens basal ganglia.
rs1634761..HLA-C..VARS2.....Brain Frontal Cortex BA9..............
rs1634761..HLA-C..VARS2.....Lung..................................
rs1634761..HLA-C..VARS2.....Pancreas..............................
rs1634761..HLA-C..VARS......Vagina................................
rs1634761..HLA-C..VWA7......Nerve Tibial..........................
IMMUNOBASE Multiple sclerosis hg19
RA GWASmeta2 20090505
UKB Basal metabolic rate
UKB Asthma
UKB Hypothyroidism or myxoedema
RA OKADA ASIAN
AdvancedAMD 2015
UKB Hyperthyroidism aka thyrotoxicosis
UKB Ankylosing spondylitis
RA OKADA EUR
RA OKADA TRANS ETHNIC
IMMUNOBASE Systemic lupus erythematosus hg19
UKB Malabsorption or coeliac disease
$P < 1x10^{-60}$

Figure S20

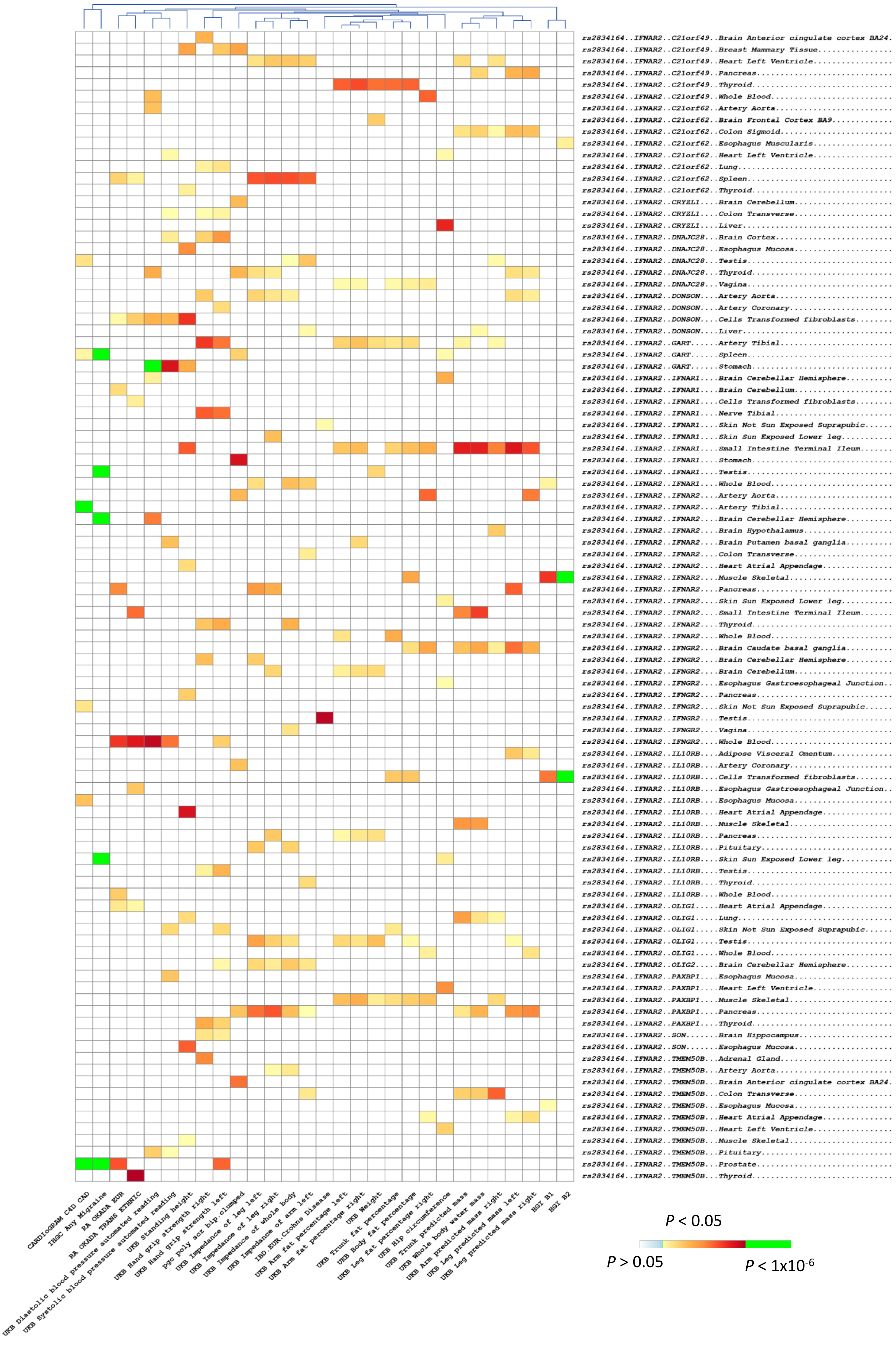

rs2834164..IFNAR2..C21orf49..Brain Anterior cingulate cortex BA24.
rs2834164..IFNAR2..C21orf49..Breast Mammary Tissue
rs2834164..IFNAR2..C21orf49..Heart Left Ventricle
rs2834164..IFNAR2..C21orf49..Pancreas
rs2834164..IFNAR2..C21orf49..Thyroid
rs2834164..IFNAR2..C21orf49..Whole Blood
rs2834164..IFNAR2..C21orf62..Artery Aorta
rs2834164..IFNAR2..C21orf62..Brain Frontal Cortex BA9
rs2834164..IFNAR2..C21orf62..Colon Sigmoid
rs2834164..IFNAR2..C21orf62..Esophagus Muscularis
rs2834164..IFNAR2..C21orf62..Heart Left Ventricle
rs2834164..IFNAR2..C21orf62..Lung
rs2834164..IFNAR2..C21orf62..Spleen
rs2834164..IFNAR2..C21orf62..Thyroid
rs2834164..IFNAR2..CRYZL1....Brain Cerebellum
rs2834164..IFNAR2..CRYZL1....Colon Transverse
rs2834164..IFNAR2..CRYZL1....Liver
rs2834164..IFNAR2..DNAJC28...Brain Cortex
rs2834164..IFNAR2..DNAJC28...Esophagus Mucosa
rs2834164..IFNAR2..DNAJC28...Testis
rs2834164..IFNAR2..DNAJC28...Thyroid
rs2834164..IFNAR2..DNAJC28...Vagina
rs2834164..IFNAR2..DONSON....Artery Aorta
rs2834164..IFNAR2..DONSON....Artery Coronary
rs2834164..IFNAR2..DONSON....Cells Transformed fibroblasts
rs2834164..IFNAR2..DONSON....Liver
rs2834164..IFNAR2..GART......Artery Tibial
rs2834164..IFNAR2..GART......Spleen
rs2834164..IFNAR2..GART......Stomach
rs2834164..IFNAR2..IFNAR1....Brain Cerebellar Hemisphere
rs2834164..IFNAR2..IFNAR1....Brain Cerebellum
rs2834164..IFNAR2..IFNAR1....Cells Transformed fibroblasts
rs2834164..IFNAR2..IFNAR1....Nerve Tibial
rs2834164..IFNAR2..IFNAR1....Skin Not Sun Exposed Suprapubic
rs2834164..IFNAR2..IFNAR1....Skin Sun Exposed Lower leg
rs2834164..IFNAR2..IFNAR1....Small Intestine Terminal Ileum
rs2834164..IFNAR2..IFNAR1....Stomach
rs2834164..IFNAR2..IFNAR1....Testis
rs2834164..IFNAR2..IFNAR1....Whole Blood
rs2834164..IFNAR2..IFNAR2....Artery Aorta
rs2834164..IFNAR2..IFNAR2....Artery Tibial
rs2834164..IFNAR2..IFNAR2....Brain Cerebellar Hemisphere
rs2834164..IFNAR2..IFNAR2....Brain Hypothalamus
rs2834164..IFNAR2..IFNAR2....Brain Putamen basal ganglia
rs2834164..IFNAR2..IFNAR2....Colon Transverse
rs2834164..IFNAR2..IFNAR2....Heart Atrial Appendage
rs2834164..IFNAR2..IFNAR2....Muscle Skeletal
rs2834164..IFNAR2..IFNAR2....Pancreas
rs2834164..IFNAR2..IFNAR2....Skin Sun Exposed Lower leg
rs2834164..IFNAR2..IFNAR2....Small Intestine Terminal Ileum
rs2834164..IFNAR2..IFNAR2....Thyroid
rs2834164..IFNAR2..IFNAR2....Whole Blood
rs2834164..IFNAR2..IFNGR2....Brain Caudate basal ganglia
rs2834164..IFNAR2..IFNGR2....Brain Cerebellar Hemisphere
rs2834164..IFNAR2..IFNGR2....Brain Cerebellum
rs2834164..IFNAR2..IFNGR2....Esophagus Gastroesophageal Junction..
rs2834164..IFNAR2..IFNGR2....Pancreas
rs2834164..IFNAR2..IFNGR2....Skin Not Sun Exposed Suprapubic
rs2834164..IFNAR2..IFNGR2....Testis
rs2834164..IFNAR2..IFNGR2....Vagina
rs2834164..IFNAR2..IFNGR2....Whole Blood
rs2834164..IFNAR2..IL10RB....Adipose Visceral Omentum
rs2834164..IFNAR2..IL10RB....Artery Coronary
rs2834164..IFNAR2..IL10RB....Cells Transformed fibroblasts
rs2834164..IFNAR2..IL10RB....Esophagus Gastroesophageal Junction..
rs2834164..IFNAR2..IL10RB....Esophagus Mucosa
rs2834164..IFNAR2..IL10RB....Heart Atrial Appendage
rs2834164..IFNAR2..IL10RB....Muscle Skeletal
rs2834164..IFNAR2..IL10RB....Pancreas
rs2834164..IFNAR2..IL10RB....Pituitary
rs2834164..IFNAR2..IL10RB....Skin Sun Exposed Lower leg
rs2834164..IFNAR2..IL10RB....Testis
rs2834164..IFNAR2..IL10RB....Thyroid
rs2834164..IFNAR2..IL10RB....Whole Blood
rs2834164..IFNAR2..OLIG1.....Heart Atrial Appendage
rs2834164..IFNAR2..OLIG1.....Lung
rs2834164..IFNAR2..OLIG1.....Skin Not Sun Exposed Suprapubic
rs2834164..IFNAR2..OLIG1.....Testis
rs2834164..IFNAR2..OLIG1.....Whole Blood
rs2834164..IFNAR2..OLIG2.....Brain Cerebellar Hemisphere
rs2834164..IFNAR2..PAXBP1....Esophagus Mucosa
rs2834164..IFNAR2..PAXBP1....Heart Left Ventricle
rs2834164..IFNAR2..PAXBP1....Muscle Skeletal
rs2834164..IFNAR2..PAXBP1....Pancreas
rs2834164..IFNAR2..PAXBP1....Thyroid
rs2834164..IFNAR2..SON.......Brain Hippocampus
rs2834164..IFNAR2..SON.......Esophagus Mucosa
rs2834164..IFNAR2..TMEM50B...Adrenal Gland
rs2834164..IFNAR2..TMEM50B...Artery Aorta
rs2834164..IFNAR2..TMEM50B...Brain Anterior cingulate cortex BA24.
rs2834164..IFNAR2..TMEM50B...Colon Transverse
rs2834164..IFNAR2..TMEM50B...Esophagus Mucosa
rs2834164..IFNAR2..TMEM50B...Heart Atrial Appendage
rs2834164..IFNAR2..TMEM50B...Heart Left Ventricle
rs2834164..IFNAR2..TMEM50B...Muscle Skeletal
rs2834164..IFNAR2..TMEM50B...Pituitary
rs2834164..IFNAR2..TMEM50B...Prostate
rs2834164..IFNAR2..TMEM50B...Thyroid
CARDIoGRAM C4D CAD
IHGC Any Migraine
RA OKADA EUR
RA OKADA TRANS ETHNIC
UKB Diastolic blood pressure automated reading
UKB Systolic blood pressure automated reading
UKB Standing height
UKB Hand grip strength right
UKB Hand grip strength left
pgc poly scz bip.clumped
UKB Impedance of leg left
UKB Impedance of leg right
UKB Impedance of whole body
UKB Impedance of arm left
IBD EUR.Crohns Disease
UKB Arm fat percentage left
UKB Arm fat percentage right
UKB Weight
UKB Trunk fat percentage
UKB Body fat percentage
UKB Leg fat percentage right
UKB Hip circumference
UKB Trunk predicted mass
UKB Whole body water mass
UKB Arm predicted mass right
UKB Leg predicted mass left
UKB Leg predicted mass right
HGI B1
HGI B2
P < 0.05
P > 0.05
P < 1x10-6

Figure S21

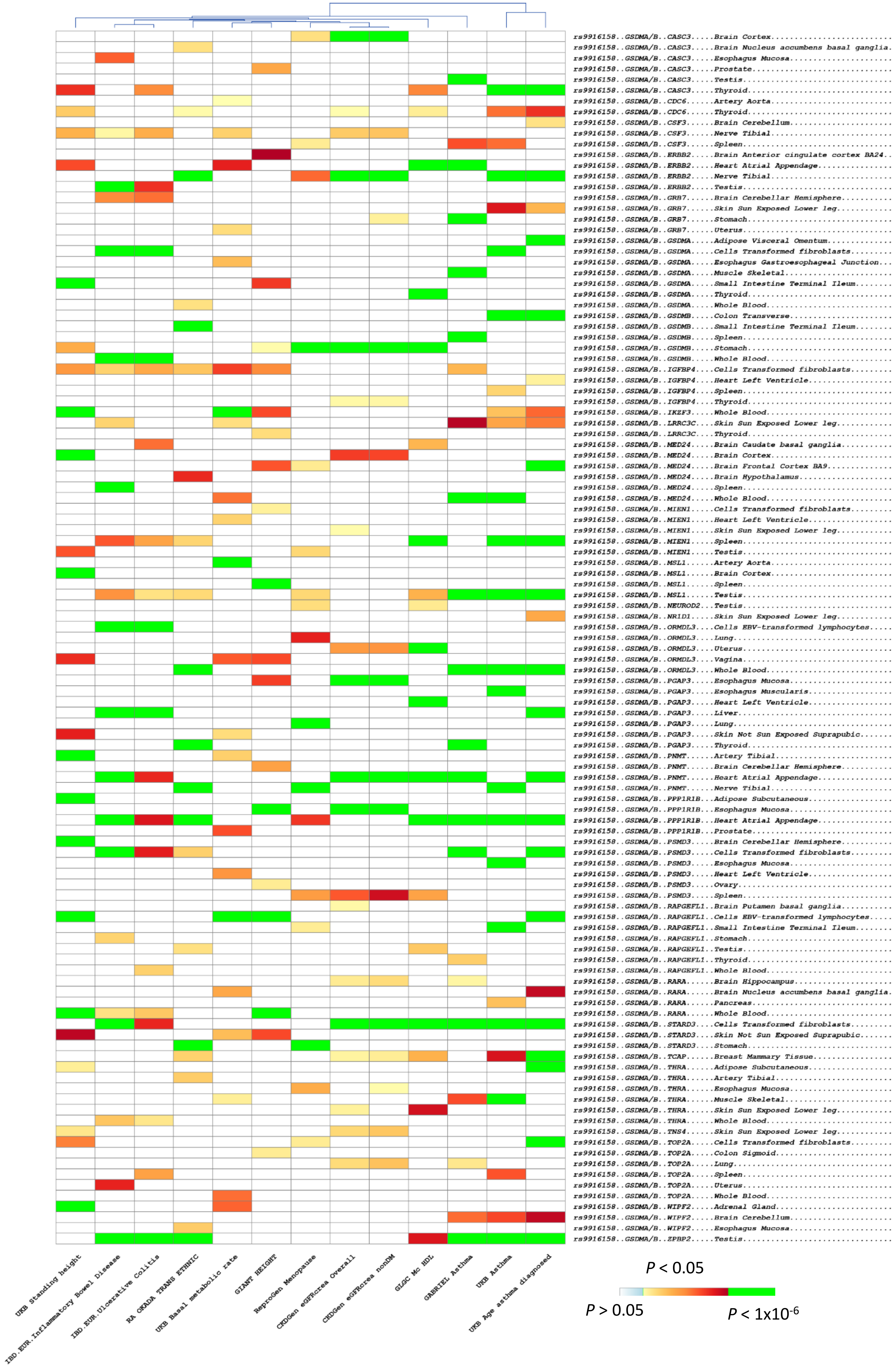

rs9916158..GSDMA/B..CASC3.....Brain Cortex
rs9916158..GSDMA/B..CASC3.....Brain Nucleus accumbens basal ganglia
rs9916158..GSDMA/B..CASC3.....Esophagus Mucosa
rs9916158..GSDMA/B..CASC3.....Prostate
rs9916158..GSDMA/B..CASC3.....Testis
rs9916158..GSDMA/B..CASC3.....Thyroid
rs9916158..GSDMA/B..CDC6......Artery Aorta
rs9916158..GSDMA/B..CDC6......Thyroid
rs9916158..GSDMA/B..CSF3......Brain Cerebellum
rs9916158..GSDMA/B..CSF3......Nerve Tibial
rs9916158..GSDMA/B..CSF3......Spleen
rs9916158..GSDMA/B..ERBB2.....Brain Anterior cingulate cortex BA24
rs9916158..GSDMA/B..ERBB2.....Heart Atrial Appendage
rs9916158..GSDMA/B..ERBB2.....Nerve Tibial
rs9916158..GSDMA/B..ERBB2.....Testis
rs9916158..GSDMA/B..GRB7......Brain Cerebellar Hemisphere
rs9916158..GSDMA/B..GRB7......Skin Sun Exposed Lower leg
rs9916158..GSDMA/B..GRB7......Stomach
rs9916158..GSDMA/B..GRB7......Uterus
rs9916158..GSDMA/B..GSDMA.....Adipose Visceral Omentum
rs9916158..GSDMA/B..GSDMA.....Cells Transformed fibroblasts
rs9916158..GSDMA/B..GSDMA.....Esophagus Gastroesophageal Junction
rs9916158..GSDMA/B..GSDMA.....Muscle Skeletal
rs9916158..GSDMA/B..GSDMA.....Small Intestine Terminal Ileum
rs9916158..GSDMA/B..GSDMA.....Thyroid
rs9916158..GSDMA/B..GSDMA.....Whole Blood
rs9916158..GSDMA/B..GSDMB.....Colon Transverse
rs9916158..GSDMA/B..GSDMB.....Small Intestine Terminal Ileum
rs9916158..GSDMA/B..GSDMB.....Spleen
rs9916158..GSDMA/B..GSDMB.....Stomach
rs9916158..GSDMA/B..GSDMB.....Whole Blood
rs9916158..GSDMA/B..IGFBP4....Cells Transformed fibroblasts
rs9916158..GSDMA/B..IGFBP4....Heart Left Ventricle
rs9916158..GSDMA/B..IGFBP4....Spleen
rs9916158..GSDMA/B..IGFBP4....Thyroid
rs9916158..GSDMA/B..IKZF3.....Whole Blood
rs9916158..GSDMA/B..LRRC3C....Skin Sun Exposed Lower leg
rs9916158..GSDMA/B..LRRC3C....Thyroid
rs9916158..GSDMA/B..MED24.....Brain Caudate basal ganglia
rs9916158..GSDMA/B..MED24.....Brain Cortex
rs9916158..GSDMA/B..MED24.....Brain Frontal Cortex BA9
rs9916158..GSDMA/B..MED24.....Brain Hypothalamus
rs9916158..GSDMA/B..MED24.....Spleen
rs9916158..GSDMA/B..MED24.....Whole Blood
rs9916158..GSDMA/B..MIEN1.....Cells Transformed fibroblasts
rs9916158..GSDMA/B..MIEN1.....Heart Left Ventricle
rs9916158..GSDMA/B..MIEN1.....Skin Sun Exposed Lower leg
rs9916158..GSDMA/B..MIEN1.....Spleen
rs9916158..GSDMA/B..MIEN1.....Testis
rs9916158..GSDMA/B..MSL1......Artery Aorta
rs9916158..GSDMA/B..MSL1......Brain Cortex
rs9916158..GSDMA/B..MSL1......Spleen
rs9916158..GSDMA/B..MSL1......Testis
rs9916158..GSDMA/B..NEUROD2...Testis
rs9916158..GSDMA/B..NR1D1.....Skin Sun Exposed Lower leg
rs9916158..GSDMA/B..ORMDL3....Cells EBV-transformed lymphocytes
rs9916158..GSDMA/B..ORMDL3....Lung
rs9916158..GSDMA/B..ORMDL3....Uterus
rs9916158..GSDMA/B..ORMDL3....Vagina
rs9916158..GSDMA/B..ORMDL3....Whole Blood
rs9916158..GSDMA/B..PGAP3.....Esophagus Mucosa
rs9916158..GSDMA/B..PGAP3.....Esophagus Muscularis
rs9916158..GSDMA/B..PGAP3.....Heart Left Ventricle
rs9916158..GSDMA/B..PGAP3.....Liver
rs9916158..GSDMA/B..PGAP3.....Lung
rs9916158..GSDMA/B..PGAP3.....Skin Not Sun Exposed Suprapubic
rs9916158..GSDMA/B..PGAP3.....Thyroid
rs9916158..GSDMA/B..PNMT......Artery Tibial
rs9916158..GSDMA/B..PNMT......Brain Cerebellar Hemisphere
rs9916158..GSDMA/B..PNMT......Heart Atrial Appendage
rs9916158..GSDMA/B..PNMT......Nerve Tibial
rs9916158..GSDMA/B..PPP1R1B...Adipose Subcutaneous
rs9916158..GSDMA/B..PPP1R1B...Esophagus Mucosa
rs9916158..GSDMA/B..PPP1R1B...Heart Atrial Appendage
rs9916158..GSDMA/B..PPP1R1B...Prostate
rs9916158..GSDMA/B..PSMD3.....Brain Cerebellar Hemisphere
rs9916158..GSDMA/B..PSMD3.....Cells Transformed fibroblasts
rs9916158..GSDMA/B..PSMD3.....Esophagus Mucosa
rs9916158..GSDMA/B..PSMD3.....Heart Left Ventricle
rs9916158..GSDMA/B..PSMD3.....Ovary
rs9916158..GSDMA/B..PSMD3.....Spleen
rs9916158..GSDMA/B..RAPGEFL1..Brain Putamen basal ganglia
rs9916158..GSDMA/B..RAPGEFL1..Cells EBV-transformed lymphocytes
rs9916158..GSDMA/B..RAPGEFL1..Small Intestine Terminal Ileum
rs9916158..GSDMA/B..RAPGEFL1..Stomach
rs9916158..GSDMA/B..RAPGEFL1..Testis
rs9916158..GSDMA/B..RAPGEFL1..Thyroid
rs9916158..GSDMA/B..RAPGEFL1..Whole Blood
rs9916158..GSDMA/B..RARA......Brain Hippocampus
rs9916158..GSDMA/B..RARA......Brain Nucleus accumbens basal ganglia
rs9916158..GSDMA/B..RARA......Pancreas
rs9916158..GSDMA/B..RARA......Whole Blood
rs9916158..GSDMA/B..STARD3....Cells Transformed fibroblasts
rs9916158..GSDMA/B..STARD3....Skin Not Sun Exposed Suprapubic
rs9916158..GSDMA/B..STARD3....Stomach
rs9916158..GSDMA/B..TCAP......Breast Mammary Tissue
rs9916158..GSDMA/B..THRA......Adipose Subcutaneous
rs9916158..GSDMA/B..THRA......Artery Tibial
rs9916158..GSDMA/B..THRA......Esophagus Mucosa
rs9916158..GSDMA/B..THRA......Muscle Skeletal
rs9916158..GSDMA/B..THRA......Skin Sun Exposed Lower leg
rs9916158..GSDMA/B..THRA......Whole Blood
rs9916158..GSDMA/B..TNS4......Skin Sun Exposed Lower leg
rs9916158..GSDMA/B..TOP2A.....Cells Transformed fibroblasts
rs9916158..GSDMA/B..TOP2A.....Colon Sigmoid
rs9916158..GSDMA/B..TOP2A.....Lung
rs9916158..GSDMA/B..TOP2A.....Spleen
rs9916158..GSDMA/B..TOP2A.....Uterus
rs9916158..GSDMA/B..TOP2A.....Whole Blood
rs9916158..GSDMA/B..WIPF2.....Adrenal Gland
rs9916158..GSDMA/B..WIPF2.....Brain Cerebellum
rs9916158..GSDMA/B..WIPF2.....Esophagus Mucosa
rs9916158..GSDMA/B..ZPBP2.....Testis
UKB Standing height
IBD.EUR.Inflammatory Bowel Disease
IBD.EUR.Ulcerative Colitis
RA OKADA TRANS ETHNIC
UKB Basal metabolic rate
GIANT HEIGHT
ReproGen Menopause
CKDGen eGFRcrea Overall
CKDGen eGFRcrea nonDM
GLGC Mc HDL
GABRIEL Asthma
UKB Asthma
UKB Age asthma diagnosed
P < 0.05
P > 0.05
P < 1x10-6

Figure S22

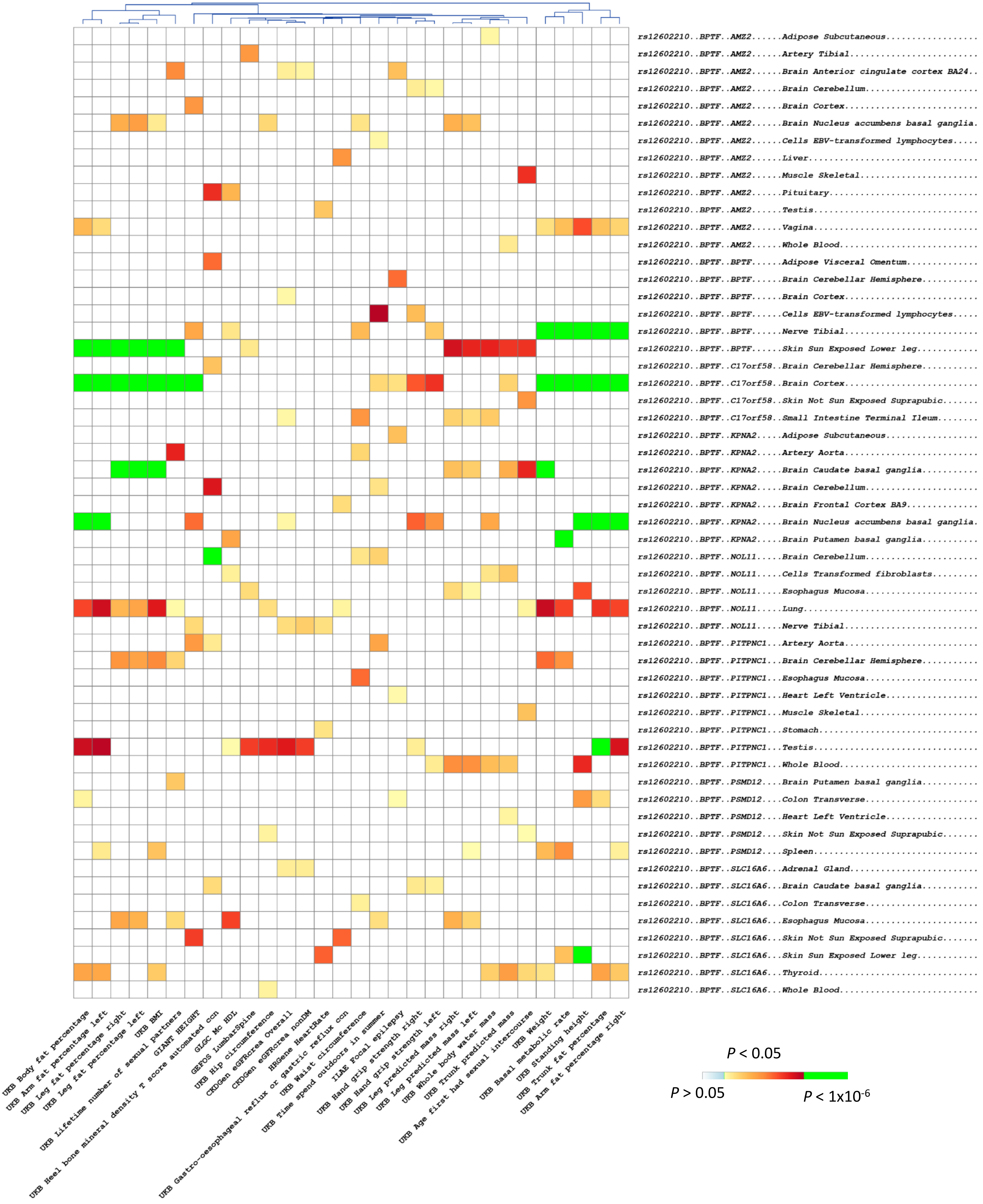

rs12602210..BPTF..AMZ2......Adipose Subcutaneous................
rs12602210..BPTF..AMZ2......Artery Tibial.......................
rs12602210..BPTF..AMZ2......Brain Anterior cingulate cortex BA24..
rs12602210..BPTF..AMZ2......Brain Cerebellum....................
rs12602210..BPTF..AMZ2......Brain Cortex........................
rs12602210..BPTF..AMZ2......Brain Nucleus accumbens basal ganglia.
rs12602210..BPTF..AMZ2......Cells EBV-transformed lymphocytes.....
rs12602210..BPTF..AMZ2......Liver...............................
rs12602210..BPTF..AMZ2......Muscle Skeletal.....................
rs12602210..BPTF..AMZ2......Pituitary...........................
rs12602210..BPTF..AMZ2......Testis..............................
rs12602210..BPTF..AMZ2......Vagina..............................
rs12602210..BPTF..AMZ2......Whole Blood.........................
rs12602210..BPTF..BPTF......Adipose Visceral Omentum.............
rs12602210..BPTF..BPTF......Brain Cerebellar Hemisphere..........
rs12602210..BPTF..BPTF......Brain Cortex........................
rs12602210..BPTF..BPTF......Cells EBV-transformed lymphocytes.....
rs12602210..BPTF..BPTF......Nerve Tibial........................
rs12602210..BPTF..BPTF......Skin Sun Exposed Lower leg...........
rs12602210..BPTF..C17orf58..Brain Cerebellar Hemisphere..........
rs12602210..BPTF..C17orf58..Brain Cortex........................
rs12602210..BPTF..C17orf58..Skin Not Sun Exposed Suprapubic......
rs12602210..BPTF..C17orf58..Small Intestine Terminal Ileum.......
rs12602210..BPTF..KPNA2.....Adipose Subcutaneous................
rs12602210..BPTF..KPNA2.....Artery Aorta........................
rs12602210..BPTF..KPNA2.....Brain Caudate basal ganglia..........
rs12602210..BPTF..KPNA2.....Brain Cerebellum....................
rs12602210..BPTF..KPNA2.....Brain Frontal Cortex BA9.............
rs12602210..BPTF..KPNA2.....Brain Nucleus accumbens basal ganglia.
rs12602210..BPTF..KPNA2.....Brain Putamen basal ganglia..........
rs12602210..BPTF..NOL11.....Brain Cerebellum....................
rs12602210..BPTF..NOL11.....Cells Transformed fibroblasts........
rs12602210..BPTF..NOL11.....Esophagus Mucosa....................
rs12602210..BPTF..NOL11.....Lung................................
rs12602210..BPTF..NOL11.....Nerve Tibial........................
rs12602210..BPTF..PITPNC1...Artery Aorta........................
rs12602210..BPTF..PITPNC1...Brain Cerebellar Hemisphere..........
rs12602210..BPTF..PITPNC1...Esophagus Mucosa....................
rs12602210..BPTF..PITPNC1...Heart Left Ventricle................
rs12602210..BPTF..PITPNC1...Muscle Skeletal.....................
rs12602210..BPTF..PITPNC1...Stomach.............................
rs12602210..BPTF..PITPNC1...Testis..............................
rs12602210..BPTF..PITPNC1...Whole Blood.........................
rs12602210..BPTF..PSMD12....Brain Putamen basal ganglia..........
rs12602210..BPTF..PSMD12....Colon Transverse....................
rs12602210..BPTF..PSMD12....Heart Left Ventricle................
rs12602210..BPTF..PSMD12....Skin Not Sun Exposed Suprapubic......
rs12602210..BPTF..PSMD12....Spleen..............................
rs12602210..BPTF..SLC16A6...Adrenal Gland.......................
rs12602210..BPTF..SLC16A6...Brain Caudate basal ganglia..........
rs12602210..BPTF..SLC16A6...Colon Transverse....................
rs12602210..BPTF..SLC16A6...Esophagus Mucosa....................
rs12602210..BPTF..SLC16A6...Skin Not Sun Exposed Suprapubic......
rs12602210..BPTF..SLC16A6...Skin Sun Exposed Lower leg...........
rs12602210..BPTF..SLC16A6...Thyroid.............................
rs12602210..BPTF..SLC16A6...Whole Blood.........................
UKB Body fat percentage
UKB Arm fat percentage left
UKB Leg fat percentage right
UKB Leg fat percentage left
UKB BMI
UKB Lifetime number of sexual partners
GIANT HEIGHT
UKB Heel bone mineral density T score automated ccn
GLGC Mc HDL
GEFOS LumbarSpine
UKB Hip circumference
CKDGen eGFRcrea Overall
CKDGen eGFRcrea nonDM
HRGene HeartRate
UKB Gastro-oesophageal reflux or gastric reflux ccn
UKB Waist circumference
UKB Time spend outdoors in summer
ILAE Focal epilepsy
UKB Hand grip strength right
UKB Hand grip strength left
UKB Leg predicted mass right
UKB Leg predicted mass left
UKB Whole body water mass
UKB Trunk predicted mass
UKB Age first had sexual intercourse
UKB Weight
UKB Basal metabolic rate
UKB Standing height
UKB Trunk fat percentage
UKB Arm fat percentage right
P < 0.05
P > 0.05
P < 1x10-6

Figure S23

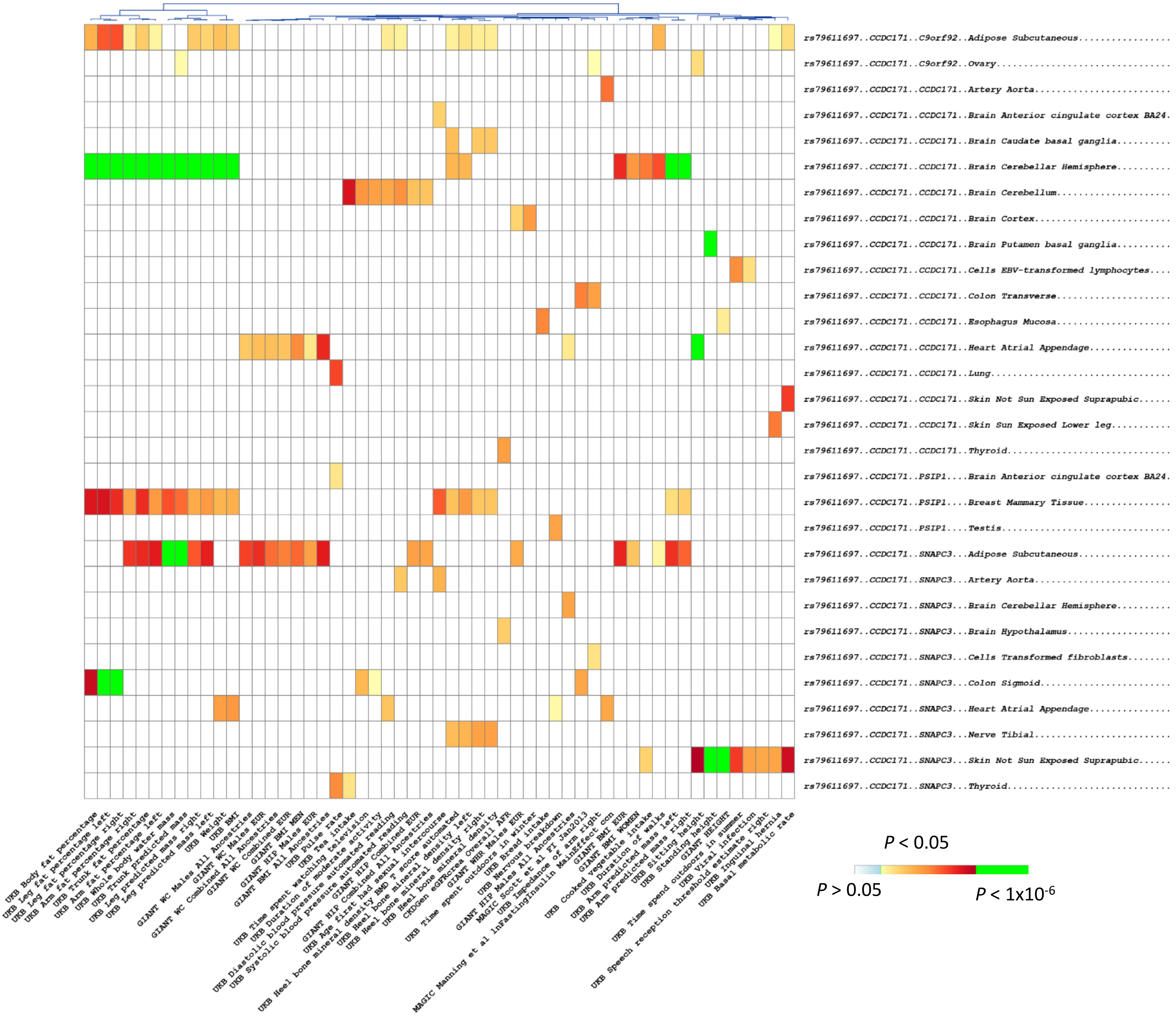
rs79611697..CCDC171..C9orf92..Adipose Subcutaneous
rs79611697..CCDC171..C9orf92..Ovary
rs79611697..CCDC171..CCDC171..Artery Aorta
rs79611697..CCDC171..CCDC171..Brain Anterior cingulate cortex BA24
rs79611697..CCDC171..CCDC171..Brain Caudate basal ganglia
rs79611697..CCDC171..CCDC171..Brain Cerebellar Hemisphere
rs79611697..CCDC171..CCDC171..Brain Cerebellum
rs79611697..CCDC171..CCDC171..Brain Cortex
rs79611697..CCDC171..CCDC171..Brain Putamen basal ganglia
rs79611697..CCDC171..CCDC171..Cells EBV-transformed lymphocytes
rs79611697..CCDC171..CCDC171..Colon Transverse
rs79611697..CCDC171..CCDC171..Esophagus Mucosa
rs79611697..CCDC171..CCDC171..Heart Atrial Appendage
rs79611697..CCDC171..CCDC171..Lung
rs79611697..CCDC171..CCDC171..Skin Not Sun Exposed Suprapubic
rs79611697..CCDC171..CCDC171..Skin Sun Exposed Lower leg
rs79611697..CCDC171..CCDC171..Thyroid
rs79611697..CCDC171..PSIP1...Brain Anterior cingulate cortex BA24
rs79611697..CCDC171..PSIP1...Breast Mammary Tissue
rs79611697..CCDC171..PSIP1...Testis
rs79611697..CCDC171..SNAPC3...Adipose Subcutaneous
rs79611697..CCDC171..SNAPC3...Artery Aorta
rs79611697..CCDC171..SNAPC3...Brain Cerebellar Hemisphere
rs79611697..CCDC171..SNAPC3...Brain Hypothalamus
rs79611697..CCDC171..SNAPC3...Cells Transformed fibroblasts
rs79611697..CCDC171..SNAPC3...Colon Sigmoid
rs79611697..CCDC171..SNAPC3...Heart Atrial Appendage
rs79611697..CCDC171..SNAPC3...Nerve Tibial
rs79611697..CCDC171..SNAPC3...Skin Not Sun Exposed Suprapubic
rs79611697..CCDC171..SNAPC3...Thyroid
UKB Body fat percentage
UKB Leg fat percentage left
UKB Leg fat percentage right
UKB Arm fat percentage right
UKB Trunk fat percentage
UKB Arm fat percentage left
UKB Whole body water mass
UKB Trunk predicted mass
UKB Leg predicted mass right
UKB Leg predicted mass left
UKB Weight
UKB BMI
GIANT WC Males All Ancestries
GIANT WC Males EUR
GIANT WC Combined All Ancestries
GIANT WC Combined EUR
GIANT BMI MEN
GIANT HIP Males EUR
GIANT BMI All Ancestries
UKB Pulse rate
UKB Tea intake
UKB Time spent watching television
UKB Duration of moderate activity
UKB Diastolic blood pressure automated reading
UKB Systolic blood pressure automated reading
GIANT HIP Combined EUR
GIANT HIP Combined All Ancestries
UKB Age first had sexual intercourse
UKB Heel bone mineral density BMD T score automated
UKB Heel bone mineral density left
UKB Heel bone mineral density right
UKB Heel bone mineral density
CKDGen eGFRcrea Overall AFR
GIANT WHR Males EUR
UKB Time spent outdoors in winter
UKB Bread intake
UKB Nervous breakdown
GIANT HIP Males All Ancestries
MAGIC Scott et al FI Jan2013
UKB Impedance of arm right
MAGIC Manning et al lnFastingInsulin MainEffect con
GIANT BMI EUR
GIANT BMI WOMEN
UKB Cooked vegetable intake
UKB Duration of walks
UKB Arm predicted mass left
UKB Arm predicted mass right
UKB Sitting height
UKB Standing height
GIANT HEIGHT
UKB Time spend outdoors in summer
UKB Viral infection
UKB Speech reception threshold estimate right
UKB Inguinal hernia
UKB Basal metabolic rate
P < 0.05
P > 0.05
P < 1x10-6

Figure S24

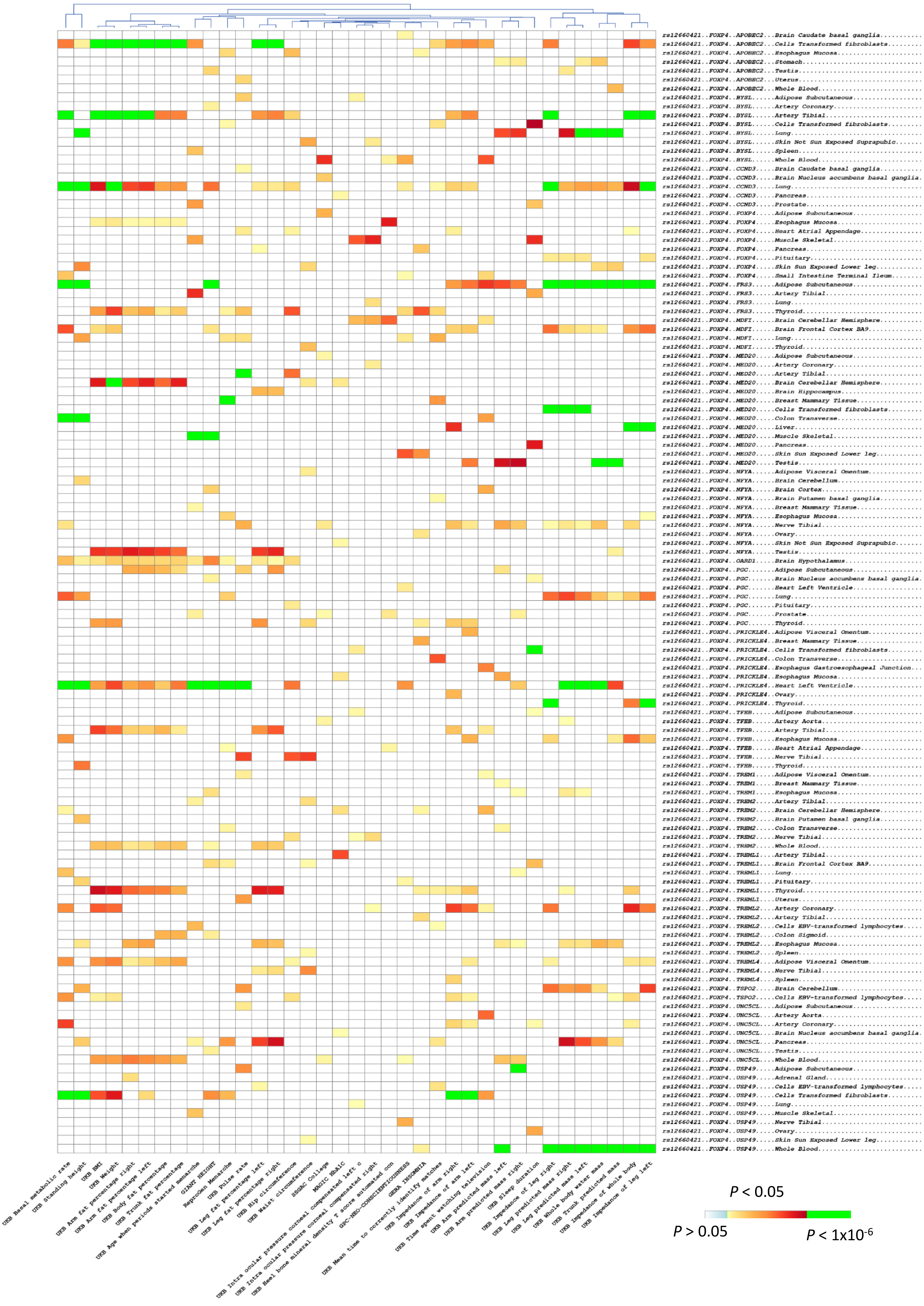

rs12660421..FOXP4..APOBEC2...Brain Caudate basal ganglia..........
rs12660421..FOXP4..APOBEC2...Cells Transformed fibroblasts........
rs12660421..FOXP4..APOBEC2...Esophagus Mucosa.....................
rs12660421..FOXP4..APOBEC2...Stomach..............................
rs12660421..FOXP4..APOBEC2...Testis...............................
rs12660421..FOXP4..APOBEC2...Uterus...............................
rs12660421..FOXP4..APOBEC2...Whole Blood..........................
rs12660421..FOXP4..BYSL......Adipose Subcutaneous.................
rs12660421..FOXP4..BYSL......Artery Coronary......................
rs12660421..FOXP4..BYSL......Artery Tibial........................
rs12660421..FOXP4..BYSL......Cells Transformed fibroblasts........
rs12660421..FOXP4..BYSL......Lung.................................
rs12660421..FOXP4..BYSL......Skin Not Sun Exposed Suprapubic......
rs12660421..FOXP4..BYSL......Spleen...............................
rs12660421..FOXP4..BYSL......Whole Blood..........................
rs12660421..FOXP4..CCND3.....Brain Caudate basal ganglia..........
rs12660421..FOXP4..CCND3.....Brain Nucleus accumbens basal ganglia.
rs12660421..FOXP4..CCND3.....Lung.................................
rs12660421..FOXP4..CCND3.....Pancreas.............................
rs12660421..FOXP4..CCND3.....Prostate.............................
rs12660421..FOXP4..FOXP4.....Adipose Subcutaneous.................
rs12660421..FOXP4..FOXP4.....Esophagus Mucosa.....................
rs12660421..FOXP4..FOXP4.....Heart Atrial Appendage...............
rs12660421..FOXP4..FOXP4.....Muscle Skeletal......................
rs12660421..FOXP4..FOXP4.....Pancreas.............................
rs12660421..FOXP4..FOXP4.....Pituitary............................
rs12660421..FOXP4..FOXP4.....Skin Sun Exposed Lower leg...........
rs12660421..FOXP4..FOXP4.....Small Intestine Terminal Ileum.......
rs12660421..FOXP4..FRS3......Adipose Subcutaneous.................
rs12660421..FOXP4..FRS3......Artery Tibial........................
rs12660421..FOXP4..FRS3......Lung.................................
rs12660421..FOXP4..FRS3......Thyroid..............................
rs12660421..FOXP4..MDFI......Brain Cerebellar Hemisphere..........
rs12660421..FOXP4..MDFI......Brain Frontal Cortex BA9.............
rs12660421..FOXP4..MDFI......Lung.................................
rs12660421..FOXP4..MDFI......Thyroid..............................
rs12660421..FOXP4..MED20.....Adipose Subcutaneous.................
rs12660421..FOXP4..MED20.....Artery Coronary......................
rs12660421..FOXP4..MED20.....Artery Tibial........................
rs12660421..FOXP4..MED20.....Brain Cerebellar Hemisphere..........
rs12660421..FOXP4..MED20.....Brain Hippocampus....................
rs12660421..FOXP4..MED20.....Breast Mammary Tissue................
rs12660421..FOXP4..MED20.....Cells Transformed fibroblasts........
rs12660421..FOXP4..MED20.....Colon Transverse.....................
rs12660421..FOXP4..MED20.....Liver................................
rs12660421..FOXP4..MED20.....Muscle Skeletal......................
rs12660421..FOXP4..MED20.....Pancreas.............................
rs12660421..FOXP4..MED20.....Skin Sun Exposed Lower leg...........
rs12660421..FOXP4..MED20.....Testis...............................
rs12660421..FOXP4..NFYA......Adipose Visceral Omentum.............
rs12660421..FOXP4..NFYA......Brain Cerebellum.....................
rs12660421..FOXP4..NFYA......Brain Cortex.........................
rs12660421..FOXP4..NFYA......Brain Putamen basal ganglia..........
rs12660421..FOXP4..NFYA......Breast Mammary Tissue................
rs12660421..FOXP4..NFYA......Esophagus Mucosa.....................
rs12660421..FOXP4..NFYA......Nerve Tibial.........................
rs12660421..FOXP4..NFYA......Ovary................................
rs12660421..FOXP4..NFYA......Skin Not Sun Exposed Suprapubic......
rs12660421..FOXP4..NFYA......Testis...............................
rs12660421..FOXP4..OARD1.....Brain Hypothalamus...................
rs12660421..FOXP4..PGC.......Adipose Subcutaneous.................
rs12660421..FOXP4..PGC.......Brain Nucleus accumbens basal ganglia.
rs12660421..FOXP4..PGC.......Heart Left Ventricle.................
rs12660421..FOXP4..PGC.......Lung.................................
rs12660421..FOXP4..PGC.......Pituitary............................
rs12660421..FOXP4..PGC.......Prostate.............................
rs12660421..FOXP4..PGC.......Thyroid..............................
rs12660421..FOXP4..PRICKLE4..Adipose Visceral Omentum.............
rs12660421..FOXP4..PRICKLE4..Breast Mammary Tissue................
rs12660421..FOXP4..PRICKLE4..Cells Transformed fibroblasts........
rs12660421..FOXP4..PRICKLE4..Colon Transverse.....................
rs12660421..FOXP4..PRICKLE4..Esophagus Gastroesophageal Junction...
rs12660421..FOXP4..PRICKLE4..Esophagus Mucosa.....................
rs12660421..FOXP4..PRICKLE4..Heart Left Ventricle.................
rs12660421..FOXP4..PRICKLE4..Ovary................................
rs12660421..FOXP4..PRICKLE4..Thyroid..............................
rs12660421..FOXP4..TFEB......Adipose Subcutaneous.................
rs12660421..FOXP4..TFEB......Artery Aorta.........................
rs12660421..FOXP4..TFEB......Artery Tibial........................
rs12660421..FOXP4..TFEB......Esophagus Mucosa.....................
rs12660421..FOXP4..TFEB......Heart Atrial Appendage...............
rs12660421..FOXP4..TFEB......Nerve Tibial.........................
rs12660421..FOXP4..TFEB......Thyroid..............................
rs12660421..FOXP4..TREM1.....Adipose Visceral Omentum.............
rs12660421..FOXP4..TREM1.....Breast Mammary Tissue................
rs12660421..FOXP4..TREM1.....Esophagus Mucosa.....................
rs12660421..FOXP4..TREM2.....Artery Tibial........................
rs12660421..FOXP4..TREM2.....Brain Cerebellar Hemisphere..........
rs12660421..FOXP4..TREM2.....Brain Putamen basal ganglia..........
rs12660421..FOXP4..TREM2.....Colon Transverse.....................
rs12660421..FOXP4..TREM2.....Nerve Tibial.........................
rs12660421..FOXP4..TREM2.....Whole Blood..........................
rs12660421..FOXP4..TREML1....Artery Tibial........................
rs12660421..FOXP4..TREML1....Brain Frontal Cortex BA9.............
rs12660421..FOXP4..TREML1....Lung.................................
rs12660421..FOXP4..TREML1....Pituitary............................
rs12660421..FOXP4..TREML1....Thyroid..............................
rs12660421..FOXP4..TREML1....Uterus...............................
rs12660421..FOXP4..TREML2....Artery Coronary......................
rs12660421..FOXP4..TREML2....Artery Tibial........................
rs12660421..FOXP4..TREML2....Cells EBV-transformed lymphocytes....
rs12660421..FOXP4..TREML2....Colon Sigmoid........................
rs12660421..FOXP4..TREML2....Esophagus Mucosa.....................
rs12660421..FOXP4..TREML2....Spleen...............................
rs12660421..FOXP4..TREML4....Adipose Visceral Omentum.............
rs12660421..FOXP4..TREML4....Nerve Tibial.........................
rs12660421..FOXP4..TREML4....Spleen...............................
rs12660421..FOXP4..TSPO2.....Brain Cerebellum.....................
rs12660421..FOXP4..TSPO2.....Cells EBV-transformed lymphocytes....
rs12660421..FOXP4..UNC5CL....Adipose Subcutaneous.................
rs12660421..FOXP4..UNC5CL....Artery Aorta.........................
rs12660421..FOXP4..UNC5CL....Artery Coronary......................
rs12660421..FOXP4..UNC5CL....Brain Nucleus accumbens basal ganglia.
rs12660421..FOXP4..UNC5CL....Pancreas.............................
rs12660421..FOXP4..UNC5CL....Testis...............................
rs12660421..FOXP4..UNC5CL....Whole Blood..........................
rs12660421..FOXP4..USP49.....Adipose Subcutaneous.................
rs12660421..FOXP4..USP49.....Adrenal Gland........................
rs12660421..FOXP4..USP49.....Cells EBV-transformed lymphocytes....
rs12660421..FOXP4..USP49.....Cells Transformed fibroblasts........
rs12660421..FOXP4..USP49.....Lung.................................
rs12660421..FOXP4..USP49.....Muscle Skeletal......................
rs12660421..FOXP4..USP49.....Nerve Tibial.........................
rs12660421..FOXP4..USP49.....Ovary................................
rs12660421..FOXP4..USP49.....Skin Sun Exposed Lower leg...........
rs12660421..FOXP4..USP49.....Whole Blood..........................
UKB Basal metabolic rate
UKB Standing height
UKB BMI
UKB Weight
UKB Arm fat percentage right
UKB Arm fat percentage left
UKB Body fat percentage
UKB Trunk fat percentage
UKB Age when periods started menarche
GIANT HEIGHT
ReproGen Menarche
UKB Pulse rate
UKB Leg fat percentage left
UKB Leg fat percentage right
UKB Hip circumference
UKB Waist circumference
SSGAC College
MAGIC HbA1C
UKB Intra ocular pressure corneal compensated left c
UKB Intra ocular pressure corneal compensated right
UKB Heel bone mineral density T score automated ccn
GPC-NEO-CONSCIENTIOUSNESS
UKB Mean time to correctly identify matches
GERA INSOMNIA
UKB Impedance of arm right
UKB Impedance of arm left
UKB Time spent watching television
UKB Arm predicted mass left
UKB Arm predicted mass right
UKB Sleep duration
UKB Impedance of leg right
UKB Leg predicted mass right
UKB Leg predicted mass left
UKB Whole body water mass
UKB Trunk predicted mass
UKB Impedance of whole body
UKB Impedance of leg left
P < 0.05
P > 0.05
P < 1x10-6
